# Molecular Dynamics Simulation of Structural Assembly and Hydration of Hyaluronic Acid in Salt Aqueous Buffer

**DOI:** 10.1021/acs.langmuir.4c03966

**Published:** 2025-02-06

**Authors:** Saranya Vasudevan, Sandipan Chattaraj, Alessandro Enrico, Francesco Silvio Pasqualini

**Affiliations:** Synthetic Physiology Lab, Department of Civil Engineering and Architecture, University of Pavia, Pavia 27100, Italy

## Abstract

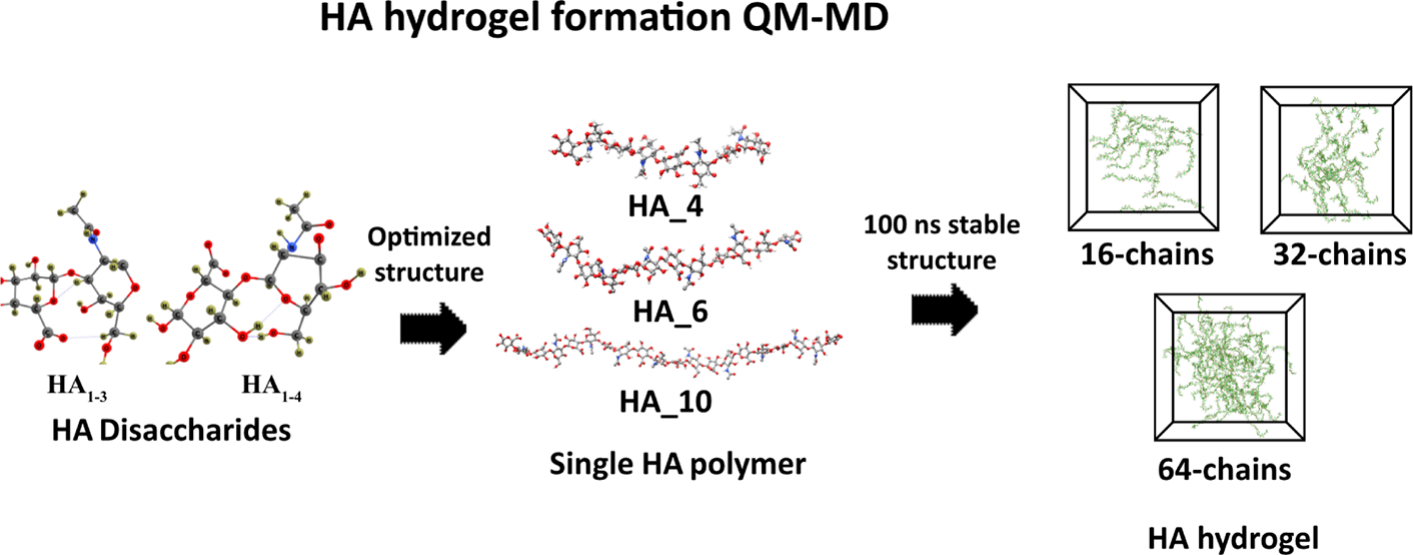

Hyaluronic acid (HA) is a nonsulfonated glycosaminoglycan critical in tissue development, physiology, and disease processes. To develop biomimetic in vitro models based on HA, it is important to understand the interaction of this polymer in its pristine form and with physiological solvents. However, atomistic simulations of HA chains are computationally challenging, especially when studying interactions with salts. To tackle this challenge, this study combined quantum mechanical (QM) calculations and molecular dynamics (MD) simulations to investigate HA’s structure and behavior. This multiscale approach balances accuracy and computational efficiency. QM calculations emphasize the role of weak noncovalent hydrogen bonds in stabilizing d-glucuronic acid with N-acetyl-d-glucosamine. MD results show that more HA layers lead to a larger structure, higher water sensitivity, and increased dynamic and interlayer complexity. Our QM and MD simulations shed light on the structural dynamics and interactions of HA polymers and HA hydrogels, aiding in their design and optimization for biomedical applications and bridging computational and experimental approaches.

## Introduction

Hyaluronic acid (HA) is a naturally occurring polysaccharide that has recently gained much attention in regenerative medicine and tissue engineering because of its crucial role in various biological processes, including cell signaling, tissue regeneration, wound healing, and pathobiology.^1 −9^ In humans, HA exists as a linear glycosaminoglycan group polymer with a 10^4^ to 10^7^ Da.^10,11^ HA production is catalyzed by class I hyaluronan synthases, while the building blocks and energy for HA polymer formations are derived from nucleotide sugars such as glucuronic acid (GlcA) and N-acetyl-d-glucosamine (GlcNAc). HA is a negatively charged polymer containing carboxyl (COO−) and hydroxyl (OH−) groups.^12^ In a physiological solution, the COO– groups within GlcA are completely ionized, forming hyaluronate anions.^11,13^ Moreover, the HA polymer chain in electrolyte solutions typically expands as a random coil configuration.^14^ Because of their biocompatibility and biodegradability, HA structures have been studied extensively over the years as they provide the fundamental basis for investigating the biological activities and functional mechanisms related to health and diseases.^3,5,10^

GlcA and GlcNAc are linked together by glycosidic linkages β_1,3_ and β_1,4_.^5,15^ In aqueous solutions, HA has a random coil formation that allows for the absorption and retention of considerable amounts of water.^16−18^ Low-molecular weight HA (repeating disaccharide unit <125 units or molecular weight <50 kDa) has a better moisturizing ability with efficient skin penetration than high-molecular weight HA.^10^ However, little has been done in the computational modeling of the interactions and structural behavior of HA under different physiological conditions. Structural insights into HA’s intrinsic flexibility are crucial to better understand the biological role of hyaluronan polymers. Because of its conformational flexibility, HA can interact with ions and other biomolecules to change its functional characteristics and structural integrity. Furthermore, the HA interaction with water molecules and salts in hydrogel formulations determines the resulting mechanical and physical characteristics, including stability and swelling behavior.^18,19^ Recent advances in molecular modeling of polysaccharides have greatly enhanced our comprehension of the structural foundations underlying the formation, stability, and interactions of highly ordered structures with counterions.^2,20^ While the mechanism of hydrogel formation has been deeply investigated in experimental research articles,^9,20,21^ there is a lack of detailed procedures for developing theoretical structural models.^9,19,21^ While existing studies focused on systems with a single salt species, a significant gap remains in our comprehensive knowledge about HA and its associated mechanisms in response to various salt environments.^22^

Because of its anionic nature, the behavior of HA in aqueous solutions is strongly influenced by pH and salt concentrations, which affect the equilibrium between the repulsive and attractive forces acting on the polymer chains.^23^ However, existing characterization methods are insufficient for assessing the precise arrangement of molecules and individual atoms at the nanometer scale, making it challenging to precisely define the hydrogel formation process and final assembly structure.^24^ Understanding the microstructure and molecular arrangement of hydrogel molecules will allow to design and alter their properties more effectively at the molecular level, thus broadening their potential applications.^25^ Previously, Furlan studied structural conformations of small oligomers,^26^ and Gargiulo performed a similar combined analysis with HA decamer through MD simulation and NMR data.^27^ Existing studies are mostly limited to very short oligomers (from disaccharides to tetrasaccharides), and they focused on a single type of salt, leaving a significant gap in our comprehensive knowledge of HA and its associated mechanisms in response to various salt environments.^22^ To tackle this challenge, this study combined quantum mechanical (QM) calculations and molecular dynamics (MD) simulations to investigate HA’s structure and behavior.^28^ This multiscale approach balances accuracy and computational efficiency. Our study focuses on three different main areas: (1) modeling HA chains of varying lengths, (2) preparing HA hydrogels, and (3) investigating the influence of various salts and different water models. By addressing these issues, this study aims to understand the HA properties and provide a more comprehensive analysis of its structural behavior, especially in biomedical applications. QM calculations can be used to optimize the structure of HA molecules and obtain the molecular configuration with a relatively stable energy for MD simulation. Therefore, in our study, we have chosen β_1–3_ and β_1–4_ linkages of HA_1–4_ and HA_1–3_ disaccharides as the base for the MD simulation of the HA hydrogel system. Through MD simulation, the final formation state of the HA hydrogel system and the intermolecular relationship were observed. Arrangement methods and the main driving force for the gelation process were confirmed from a microscopic point of view to gain a deeper understanding of the gelation behavior of HA hydrogels. Finally, we provided the detailed preparation method for the HA hydrogel model and examined the influence of external parameters, such as water, salts, and their concentration on the HA and hydrogel structures and their behavior.

## Materials and Methods

### Quantum Chemistry Calculations

In this study, a starting block for HA models with different repeated disaccharide units and chain lengths was built based on the structure of PDB code 2BVK.^29^ All the QM calculations were done using the ORCA quantum chemistry program package,^30^ which includes different modern electronic structure methods such as density functional theory (DFT),^31,32^ coupled cluster theories, many-body perturbation, and multireference semiempirical methods. All geometries were optimized by density functional theory (DFT) using Becke, 3-parameter, Lee–Yang–Parr (B3LYP)^31−34^ functionals and the def2-SVP^7,35,36^ basis set incorporating the effective core potential (ECP). The B3LYP/def2-SVP basis sets have been effectively used in numerous biomolecular studies, producing reliable results for structural, energetic, and electronic properties.^35^ The HA_1–4_ and HA_1–3_ disaccharide structures are optimized in the gas and water phases, and no significant variations were observed between the energy values, indicating that the structure is stable and does not significantly change between the two different environments (Figure S1). To reduce the computational cost without sacrificing the accuracy, we used gas phase coordinates for all of the analyses. Figure S1A,B shows the lowest energy conformation after optimizing the β_1–3_ and β_1–4_ linkages of HA_1–4_ and HA_1–3_ disaccharides. The analytical Hessian method’s vibrational frequencies were used to calculate the zero-point-corrected energy at 0 K. The Multiwfn code^37^ was used to perform a noncovalent interaction (NCI) analysis, and the indices were computed using a 25 Å radius threshold. NCIs can be identified using an index that the NCI analysis offers, which is based on electron density and its derivatives. It is based on a 2D representation of the electron density (ρ), and the reduced density gradient (*s*, eq 1):^38^
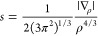
1

### Molecular Dynamics Simulation Setup

The HA hydrogel is built and analyzed to understand the influence of varying water mass fractions on hydrogel behavior. Prior to these simulations, preliminary investigations were conducted to prepare a different number of repeated HA disaccharide unit configurations, various chain lengths, and salt concentrations. Initially, a pdb file for 4 repeated disaccharides (HA_4_) was built based on the PDB code 2BVK structure, with the GlcNAc and GlcA saccharides linked by alternating β_1–3_ and β_1–4_ glycosidic linkages. A polymer of 20 repeating disaccharides (HA_10_) was built by using that structure, and all three systems were simulated for 500 ns (Figure S2). Subsequently, the resulting stable structures after 500 ns simulations were employed to further simulate the inclusion of NaCl salt with charge-neutralized concentrations (0.1 M) (Figure S2B). Afterward, these structures were used to prepare hydrogels with different numbers of HA_10_ chains (16, 32, and 64 chains). The GROMACS “insert-molecules” tool is used to insert multiple HA chains in the box, which randomly distribute the molecules in the simulation box while ensuring that they do not overlap with existing molecules. For our study, we chose 16, 32, and 64 chains for HA hydrogel configurations to examine various network complexities and their effects on their properties. To understand the fundamental interaction while being computationally efficient, we used simpler networks of the 16 chains. Meanwhile, 32 and 64 chain configurations shed light on the impact of moderate and high polymer density on hydrogel properties and to identify effects at high network density. All MD simulations were performed using the GROMACS 2022^39,40^ package with Chemistry at HARvard Molecular Mechanics (CHARMM) force field.^41^ The CHARMM force field is vital for bio and organic molecule simulations because it accurately and comprehensively signifies molecular interactions.^41^

Periodic boundary conditions were imposed in all directions, and the LINear Constraint Solver (LINCS) algorithm was applied to all bonds. To maintain the balance between computational efficiency and accuracy, the cutoff value for nonbonded interactions was set at 11.32 Å. Longer-range interactions are managed by using the particle-mesh Ewald (PME) method. MD trajectories were integrated using a leapfrog integrator with a time step of 1 fs, and snapshots were saved for every picosecond. To evaluate the effect of water molecules on the hydrogel, we examined eight hydrated systems made up of three distinct HA models (16, 32, and 64 chains). Afterward, various water content models (10%, 40%, 60%, 80%, 90%, 95%, 99%, and 99.99%) were introduced in the simulation box using the TIP3P water molecule model.^42^ We defined and calculated the percentage of the water models as the ratio between the number of atoms from water molecules and the number of atoms from HA, which reflects the experimental preparation (Table S1). For lower water concentration systems (10% water model), we used the isobaric–isothermal ensemble (NPT) ensemble. Berenden’s temperature coupling method was used to maintain the system temperature at 323 K, and the Parrinello–Rahman method was used to control the pressure at 1 bar. The periodic boundary conditions (PBC) were used consistently throughout the simulations to reduce edge effects, ensure that the system remained realistic, and avoid potential artifacts. As mentioned above, the water content models are defined based on the total mass of HA chains, allowing us to study their influence on the formation and stability of the HA hydrogel. The same box size was used for all simulations to maintain consistency and simplify comparisons between the different systems. While this approach ensures that the concentration does not directly affect the HA chains themselves, it allows us to observe how concentration differences might influence the overall system properties. 0.1 M NaCl was added to the aqueous solution to reach physiological ionic strength. In both the NVT and NPT ensembles, the leapfrog integration technique with a time step of 0.5 fs was used for equilibration and reduction. All MD simulations were run for three distinct trials of 100 ns. The script files for MD simulation setup and polymer analysis script files for HA polymers are attached to the Supporting Information material and can be used to reduce the system preparation process. This approach reduces the preparation time by approximately 30–50%, ensuring high accuracy and efficiency in modeling complex polymer systems.

To investigate the hydrodynamic properties of the HA hydrogel and the diffusion behavior of water molecules within it, mean square displacements (MSD) and the diffusion coefficients (D) are computed.^43^ The calculation of MSD was carried out using eq 2:
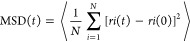
2where the angle brackets signify the ensemble average, *N* represents the number of water molecules, and *ri*(*t*) and *ri*(0) denote the final and initial positions of the water molecular mass center at time *t*. The diffusion coefficients (*D*) are calculated using Einstein’s eq (eq 3):
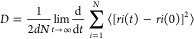
3where *d* represents the dimensionality of the system.

## Results

### Quantum Mechanical Calculations: Identification and Impact of Noncovalent Interactions on HA Polymer and Stability

**Figure 1:**
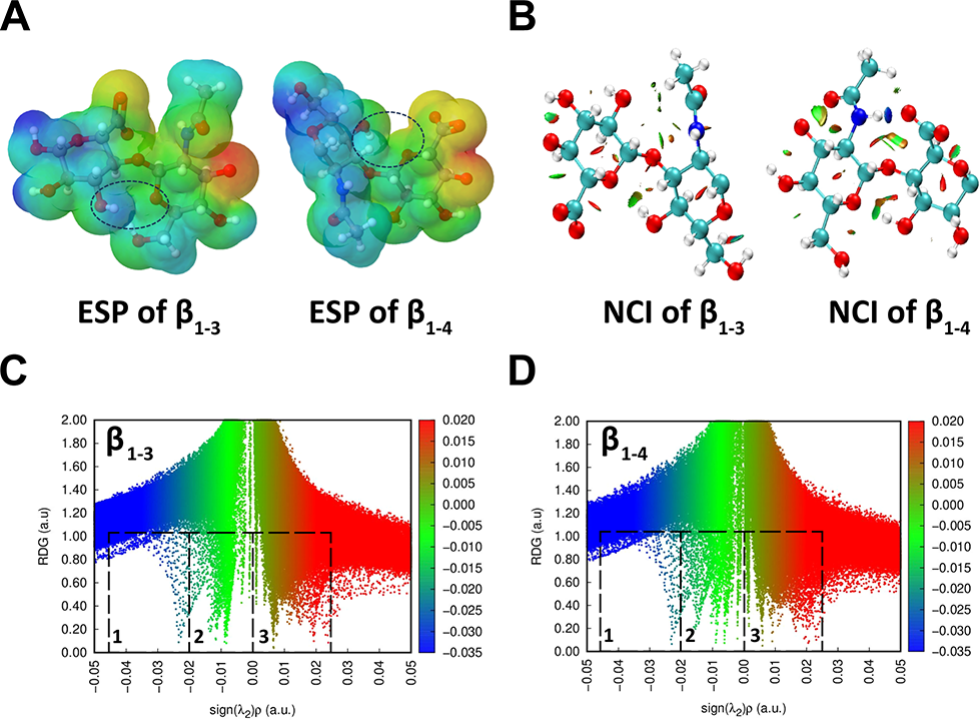
Quantum mechanical calculations for GlcA and GlcNAc. (A) Electrostatic potential (ESP) map of the β_1–3_ and β_1–4_ linkages of HA_1–3_ and HA_1–4_ disaccharides. The dotted circles highlight two hydrogen bonding interactions. (B) 3D noncovalent interaction (NCI) isosurface plots. Carbon atoms are represented in cyan, nitrogen in blue, O in red, and hydrogen in white. (C and D) Reduced density gradient (RDG) plots of β_1–3_ and (b) β_1–4_ linkage disaccharides, respectively. Regions 1, 2, and 3 highlight the density ranges affected by hydrogen bonding, vdW forces and π···π stacking, and steric repulsion, respectively.

We performed QM calculations to produce small-scale maps of molecular electrostatic potential (ESP) and noncovalent interactions (NCI) for the disaccharide units of HA (Figure 1 and S1). Since these interactions are essential to maintain the structural stability and integrity of HA molecules, we analyzed electron density (ED) distributions to better understand and control the chemical, physical, and biological properties of HA molecules. The hydrophilic and hydrophobic regions of HA disaccharides can be examined using ESP maps (Figure 1A).^44^ Hydrophilic regions show a negative electrostatic potential, while hydrophobic parts show a positive or less negative electrostatic potential than that from hydrophilic regions. The different regions are highlighted on ESP maps using different color coding: zero potential is indicated by green, blue denotes the positive region preferred for nucleophilic attack, and red denotes the negative region favored for electrophilic attack. In Figure 1A, an isosurface diagram illustrates HA disaccharides, depicting a negative charge area enveloping the O atom of GlcNAc and the carboxylic oxygen atoms of GlcA, which serve as hydrogen acceptors.^45^ Moreover, a positive charge density surrounds the hydroxyl groups of GlcA and C=O–C–H_2_ of GlcNAc, acting as hydrogen donors. In addition to covalent bonding interactions, weak NCIs, such as van der Waals (vdW) attractive forces, π···π stacking, and hydrogen bonding, are essential to hold GlcA and GlcNAc together, which greatly adds to the stability of the disaccharides.

RDG plots and the product of the electron density (Sign(λ_2_)ρ) and the sign of the second eigenvalue of the Hessian matrix (λ_2_) are shown in Figure 1B–D, respectively. These illustrations are essential for understanding different aspects of NCI. NCI analysis, denoted by eq 1, is a more comprehensive framework for interpreting NCI in molecular systems that makes use of sign(λ_2_)ρ as well as the reduced density gradient (RDG). To be more precise, these factors are combined by the NCI approach to determine areas of attractive and repulsive interactions. This critical pair of functions is referred to as RDG. To differentiate between the several kinds of weak interactions seen in the disaccharide, such as hydrogen bonding, steric repulsion, π···π stacking, and vdW interactions, we employed the second derivative of the density. We used averaged NCI (aNCI), an extended NCI method that combines data from several frames to analyze weak interactions across the HA disaccharides.^38^ In low-density areas, as seen on the left side of Figure 1C,D, eq 1 approaches zero, displaying three spikes in the RDG plot inside the optimized compound. These spikes correspond to weak vdW attractions (effective density range: −0.020 to 0.000, including π···π stacking interactions) and hydrogen bonding (effective density range: −0.045 to −0.020), as well as steric crowding around 0.000–0.025 areas. Similarly, averaged 0 effective density is surrounded by three different spikes in the aRDG plot. The nonseparable hydrogen bonds and vdW interactions are indicated by the negative density regions, while positive density regions suggest steric repulsion.

To further explore this matter, a detailed analysis of the 3D RDG distribution and potential energy surface was conducted (Figure 1B). For both the β_1–4_ linkage and the β_1–3_ linkage of HA, the RDG plot shows clear spikes in the low-density region, suggesting the presence of different weak NCIs such as π···π stacking, vdW forces, hydrogen bonding, and steric repulsion. There are three unique contact zones, each serving a special role. Blue regions indicate the presence of hydrogen bond formation, green zones represent vdW interactions, and red zones represent repulsive interactions, which occur mostly at the cyclic level. Figure 1A shows two hydrogen bonding interactions in the optimized structure (O_6_–H_16_···O_32_ and O_34_–H_38_···O_6_), illustrated by 3D blue-green isosurfaces. Our QM findings shed more light on the chemical relationships and energy profiles of HA polymers. Strong and weak interaction regions are highlighted by analyzing the RDG distribution, which shows noncovalent interaction regions of the HA disaccharides. The observed electrostatic potential energy surface map elucidates the charge distribution, molecular diffusion, and molecular interaction sites that govern how disaccharides provide insights into molecular diffusion and rearrangement within the hydrogel, with implications for drug delivery applications.^6,46,47^ When taken as a whole, these QM findings clarify the energy landscape of the HA disaccharides as well as the spatial heterogeneity in molecular interactions. This method can shed light on the stability and reactivity of HA disaccharides under diverse circumstances, clarifying its dynamic behavior and performance in a range of applications.

### Molecular Dynamics Results for Hyaluronic Acid

#### Structural Properties of Short (HA_4_ and HA_6_) and Long Chain HA (HA_10_) Polymers

Root-mean-square deviation (RMSD) analyzes the average distance between atoms in a structure at a given point in the simulation against a reference initial structure.^48^Figure S2A shows the RMSD of HA_4_, HA_6_, and HA_10_ systems for NaCl salt with 0.1 M concentration. The calculated RMSD values for various repeating HA disaccharides serve as an important benchmark for measuring the convergence of HA systems. The conformational structure of the HA_4_, HA_6_, and HA_10_ system for a 500 ns simulation is shown in Figure S2B. Throughout the simulations, all simulated HA polymer systems demonstrated the expected aim of stability. The radius of gyration (Rg) analysis quantifies the compactness of the HA polymer and shows how it spreads out in space.^49^ The Rg results show that the overall structure of the HA polymer is stable (Figure S2C). The mean Rg values for the HA_4_, HA_6_, and HA_10_ systems were found to be 1.15, 1.6, and 2.7 nm, respectively. This result agrees with the previous findings, which suggest that the degree of fluctuations in RMSD and Rg values apparently increased as the size of HA increased.^10^ To further understand the molecular flexibility of the HA chain, root-mean-square fluctuations (RMSF) have been calculated, as shown in Figure S2D. For all the systems, we notice a general pattern of larger RMSF values at the terminal residues, indicating more flexibility at the end terminal regions of the HA polymer.^13^ In order to create a hydrogel system for further research, we used the stable structure of HA_10_.

To study the influence of various salts on the HA chain, we initially simulated the HA_6_ system for various salts (NaCl, KCl, CaCl_2_) and different concentrations of charge: neutralized state, 0.1 M, 0.5 M, and 1 M. The Rg plot for HA_6_ for various salts (NaCl, KCl, CaCl_2_) in the charge-neutralized state and for different concentrations of 0.1 M, 0.5, and 1 M is shown in Figure S3. The Rg of the charge-neutralized system varies approximately 1.6 nm for the NaCl and KCl systems, 1.7 nm for CaCl_2_ with more pronounced changes, and slightly less for KCl. Both NaCl and KCl have an Rg of about 1.6 nm at 0.1 M concentration, with NaCl showing more noticeable oscillations than in the charge-neutralized system. When compared to the charge-neutralized system, Rg for the 0.1 M CaCl_2_ system shows higher fluctuation around 1.6 nm with more noticeable variations. The CaCl_2_ system has an Rg of 1.6 to 1.7 nm, which improves stability over 0.5 M but is still less stable than NaCl and KCl. The Rg of HA was significantly higher only at 1 M concentration, indicating the greatest structural deviation at this concentration. In contrast, at all other concentrations, Rg remained consistently low, suggesting a more compact and stable structure. The hydrogen bonds are formed between water molecules and the HA chains. Moreover, the presence of Ca^2+^ can alter the balance between hydrogen bonding and ionic interactions, leading to changes in the water distribution and chain conformation. These electrostatic disturbances can either enhance or hinder the formation of hydrogen bonds between HA molecules depending on the local ionic environment.

**Figure 2:**
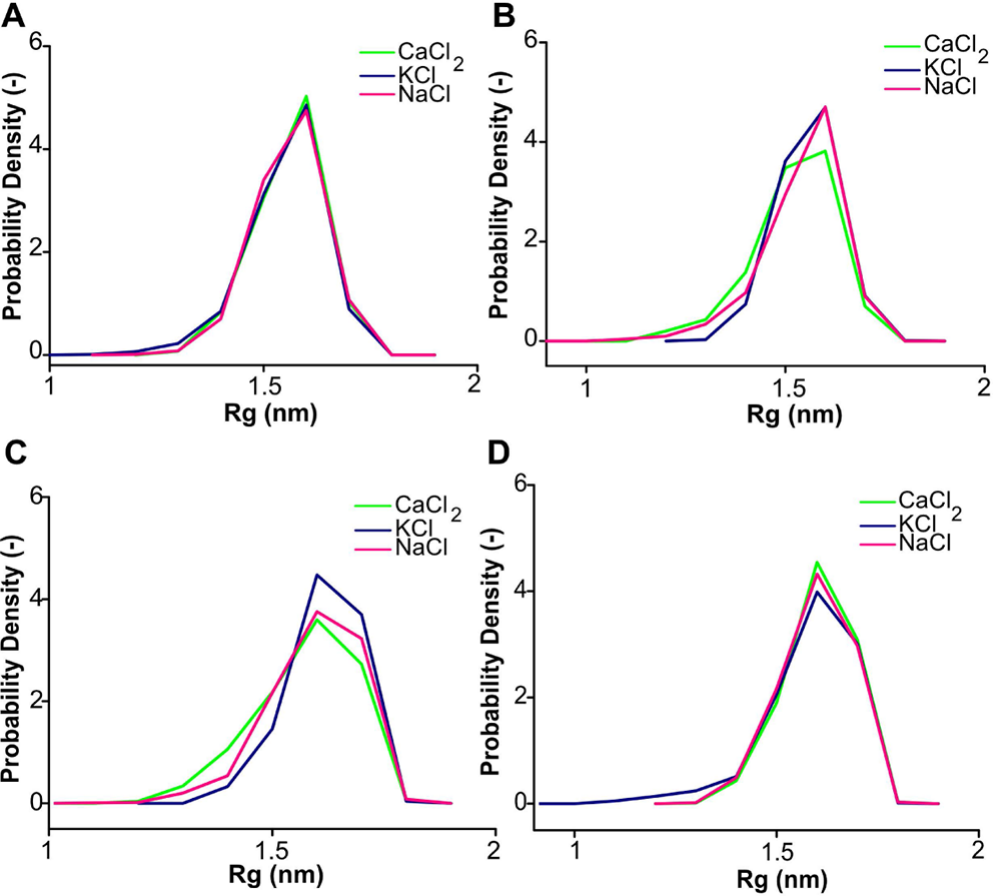
Distribution of Rg for 6 repeated HA units (HA_6_) at (Å) charge-neutralized state and (B) 0.1, (C) 0.5, and (D) 1 M concentrations.

The mean Rg along with the standard deviations calculated for 6 repeated HA disaccharide units (HA_6_) at different concentrations is shown in Figures 2 and S4. The mean value of Rg gradually increased with increasing salt concentration, and this result aligns well with the previous finding that the HA_6_ becomes less stable with increasing cation concentration.^22^ In a charge-neutralized state and at low salt concentrations (0.1 M), the HA_6_ experiences a stronger electrostatic repulsion between the ion and its negatively charged carboxylate groups, which leads to a more compact and stable structure. At higher salt concentrations (0.5 and 1 M), this electrostatic repulsion is neutralized by the ion binding. Consistent with this finding, previous studies also suggest that the presence of Ca^2+^, Na^+^, and K^+^ causes a reduction in HA chain stiffness.^50^

In order to understand the influence of cations and anions on the HA molecule structure, the distribution of cations and anions has been calculated (Figures S5 and S6). In the case of cation distribution around HA molecules, Ca^2+^ has the highest peak due to its higher charge density and smaller size, which leads to stronger interactions with HA molecules compared to the larger, less charged Na^+^ and K^+^ cations, resulting in lower peaks for NaCl and KCl. For Cl^–^ ions, the distribution is higher for CaCl_2_ near the HA hydrogel surface, mainly due to the increased density of Ca^2+^ ions around the HA hydrogel molecules, which increases the surface net charge and attracts Cl^–^ ions through electrostatic interaction. NaCl and KCl systems show lower peaks for Cl^–^ due to weaker electrostatic interactions between Na^+^ or K^+^ and HA hydrogel molecules. This behavior reflects the influence of the ion size, charge density, and electrostatic interactions in the distribution of ions in the system. When compared to other salts, such as CaCl_2_, the variations in Rg with NaCl are less pronounced, indicating that NaCl has a smaller impact on the HA structure, preserving its integrity and functional qualities more steadily over time. NaCl is used as a common physiological salt, making hydrogels in NaCl solutions more relevant for biomedical applications. The presence of NaCl in polymer and hydrogel systems preserves the hydrogels primary structure and function while avoiding substantial chemical changes or cross-linking.^51^ This feature led to the utilization of this NaCl-interacting HA structure in our HA hydrogel preparation setup.

#### Investigation of Structural Flexibility in HA Chain

The average atomic mobility of each residue in the HA polymer is measured using the root-mean-square fluctuation (RMSF), which sheds light on the stability and flexibility of various molecular regions.^52^ The RMSF was calculated for every residue in the 6 repeating HA disaccharide systems (HA_6_) (Figure S7). In all the systems, we find a general trend of larger RMSF values at the terminal residues (positions 1 and 12), which specifies increased flexibility at the terminal regions, in good agreement with the previous theoretical study.^13^ In the polymer structure, the terminal residues exhibit more mobility.^13^ Lower RMSF values are generally found in the center residues, indicating a more rigid core structure. The RMSF values for the different salts and concentrations differ slightly from one another in the graphs. The graphs reveal subtle differences in the RMSF profiles among the various salt conditions and concentrations. As expected, the RMSF profile is relatively steady across the different salts under charge-neutralized conditions. The RMSF patterns vary as the concentration of salt increases, suggesting that different concentrations of salts have distinct effects on the dynamics of the polymer. At higher concentrations (0.5 and 1 M), CaCl_2_ system shows higher RMSF values in some residues compared to KCl and NaCl, mostly in the central regions of the HA. Some residues exhibit greater variations with higher salt concentrations, whereas others remain relatively constant or even exhibit decreased mobility. The effect of the salt concentration is not consistent for all residues. Due to the presence of charged regions in those places, the salts may interact with the residues in a particular way. Remarkably, KCl and NaCl RMSF profiles are similar to one another, especially at lower concentrations, while CaCl_2_ exhibits a distinct behavior due to its divalent nature. The RMSF graphs offer significant insights into how varying concentrations and solutions of salt impact the HA structure’s local stability and flexibility. These findings are crucial for understanding polymer-salt interactions, which can significantly impact HA polymer stability, function, and behavior under a wide range of physiological and experimental conditions.

##### Structural Properties of HA Hydrogel

**Figure 3:**
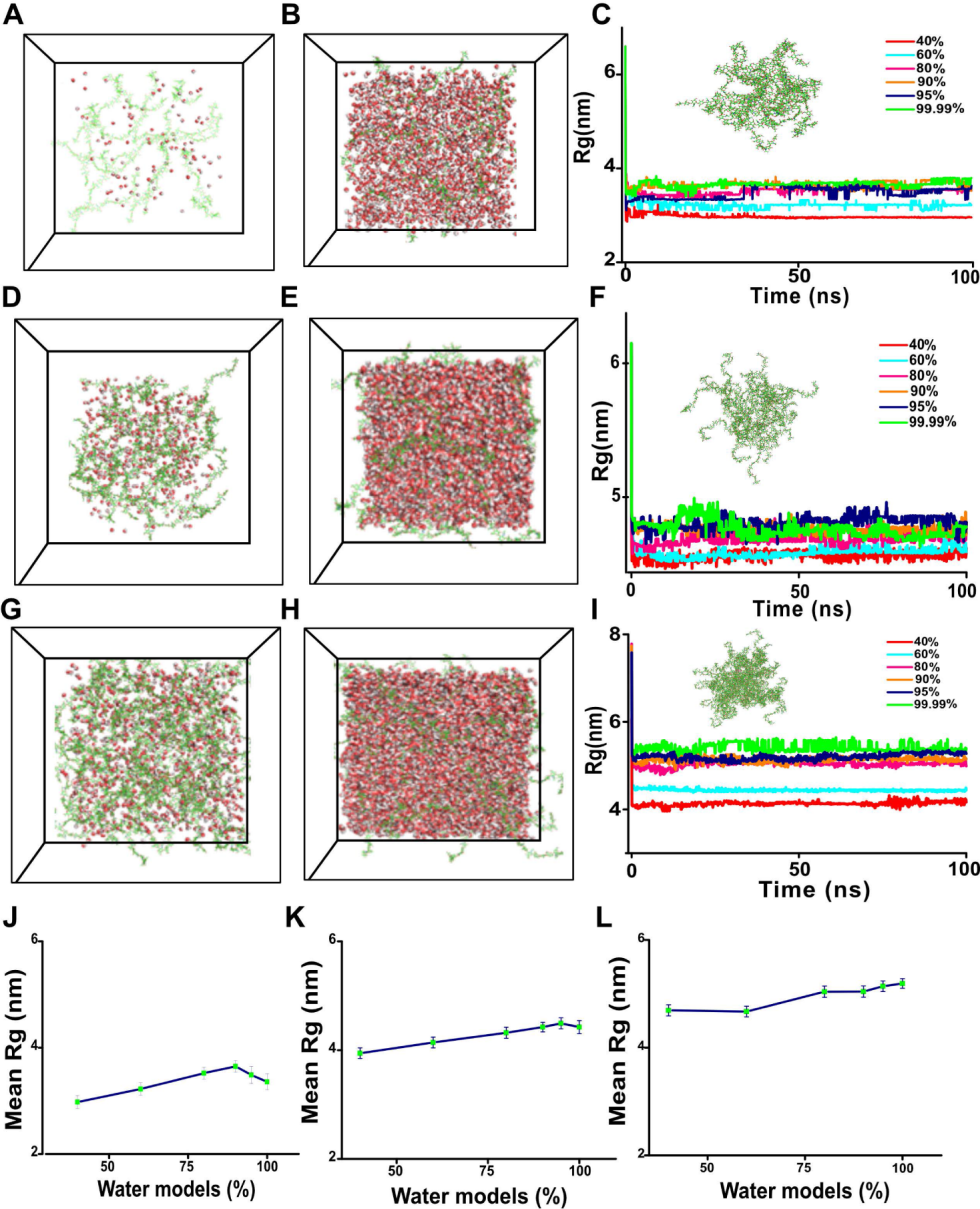
Structural properties of the HA hydrogel. (A, D, and G) The initial structure of HA chains for the lowest water model (10%). (B, E, and H) The initial structure of HA for the highest water percentage (99.99%). (C, F, and I) The radius of gyration for HA hydrogel for different water models for 16 chains, 32 chains, and 64 chains, respectively. (J–L) Mean Rg along with standard deviations of HA hydrogel for different water models for 16 chains, 32 chains, and 64 chains, respectively. The percentage of water models is calculated as defined in the Molecular Dynamics Simulation Setup, and the exact atom counts are listed in Table S1.

Our previous examination of individual HA chains found significant stability, with an average Rg of 1.6 nm and an RMSF ranging from 2 to 5 Å. The mobility of the carboxyl groups was found to be greater than that of the glycosidic connections, indicating their possible involvement in interchain interactions (Figure S8). These insights are important for individual HA units, but HA in practical applications frequently occurs in complex configurations, particularly in hydrogels. Next, we examined the effects of stacking multiple HA chains on the overall structural and dynamic properties of HA hydrogels to bridge the gap between single-chain behavior and bulk hydrogel properties. It is similar to how HA hydrogels normally form when individual chains combine to form a dense network. Initially, three different HA hydrogel systems were prepared by adding 16, 32, and 64 chains of HA in different water content models (10–99.99%); however, for the analysis, we selected only models with 40%, 60%, 80%, 90%, 95%, and 99.99% models, as shown in Figure 3A–I. The system with 16 chains (Figure 3C) exhibits the lowest Rg values, while the systems with 32 chains (Figure 3F) and 64 chains (Figure 3I) exhibit the highest Rg values. The mean Rg along with the standard deviations calculated for 16, 32, and 64 chains of HA in different water content models are shown in Figure 3J–L. Notably, the Rg values of the 16-chain systems range between 2.8 and 3.8 nm, the 32-chain systems between 4.0 and 4.4 nm, and the 64-chain systems between 4.5 and 6.5 nm. When the number of chains increases, Rg increases nonlinearly. At high water levels (90–99.99%), the 16-chain system exhibits saturation with the least Rg variations, and it is the most stable system overall with few changes.

The 32-chain system exhibits moderate stability with some fluctuations, especially at higher water contents, and achieves relative stability after initial reorganization. In contrast, the 64-chain system is the least stable, exhibiting significant fluctuations, especially at 60% and 80% water content, and continues to show these variations, indicating ongoing structural rearrangements. Water content effects are also more pronounced in the 32- and 64-chain systems: the 16-chain system shows minimal differences in Rg at high water contents (90–99.99%), and the 32- and 64-chain systems display more distinct separation between different water contents, indicating greater sensitivity to hydration levels. For models with higher water content, the 16- and 32-chain systems are relatively stable with minimum fluctuations. On the other hand, the 64-chain system is the least stable, showing notable fluctuations, particularly at 60% and 80% water models. The 32- and 64-chain systems exhibit greater sensitivity to hydration levels due to their more distinct separation between different water contents, while the 16-chain system shows minimal differences in Rg at high water contents (90–99.99%). These water content effects are also more noticeable in the 32- and 64-chain systems.

Compared with the other systems, the 16-chain system stabilizes at all water contents rapidly, the 32-chain system stabilizes somewhat after the first reorganization, and the 64-chain system exhibits considerable oscillations that imply continuous structural reorganizations. The complexity and dynamic nature of the hydrogel structure increase with the number of chains. HA hydrogels have been demonstrated in numerous studies to swell dramatically with increasing water content.^12,13,32^ HA is, in fact, extremely hygroscopic and capable of absorbing considerable volumes of water, which causes the hydrogel network to expand and swell.^20^ The increasing Rg with a higher water content aligns well with this observation. It is observed that an increase in the number of chains results in a larger overall structure, more complex interchain interactions, and higher Rg values that are particularly sensitive to water. Hydrogels with a higher water content often have higher polymer chain mobility and solute diffusion rates.^20^ This is due to the increased free volume and reduced friction between polymer chains in a more hydrated network.

Higher Rg fluctuations at higher water percentages indicate HA polymer chain mobility, in accordance with experimental findings that demonstrate increased mobility and diffusion rates in highly hydrated hydrogels.^15,33^ Previous works in rheological and dynamic light scattering characterizations of hydrogels have also found that these systems exhibit more dynamic structural behavior as water content increases, with larger and more frequent fluctuations in network dimensions.^16,17,20,53^ The intra- and intermolecular hydrogen bonds between HA chains and water molecules in various water content models are calculated (Figure S9). It is shown that when water content increases, the number of hydrogen bonds formed between HA and water molecules increases, while the number of hydrogen bonds formed between HA molecules gradually decreases. These hydrogen bonds allow the chains to connect together and eventually result in a stable three-dimensional network structure.^28,54^

#### Molecular Mobility in HA Hydrogels: A Mean Square Displacement (MSD) Study

**Figure 4:**
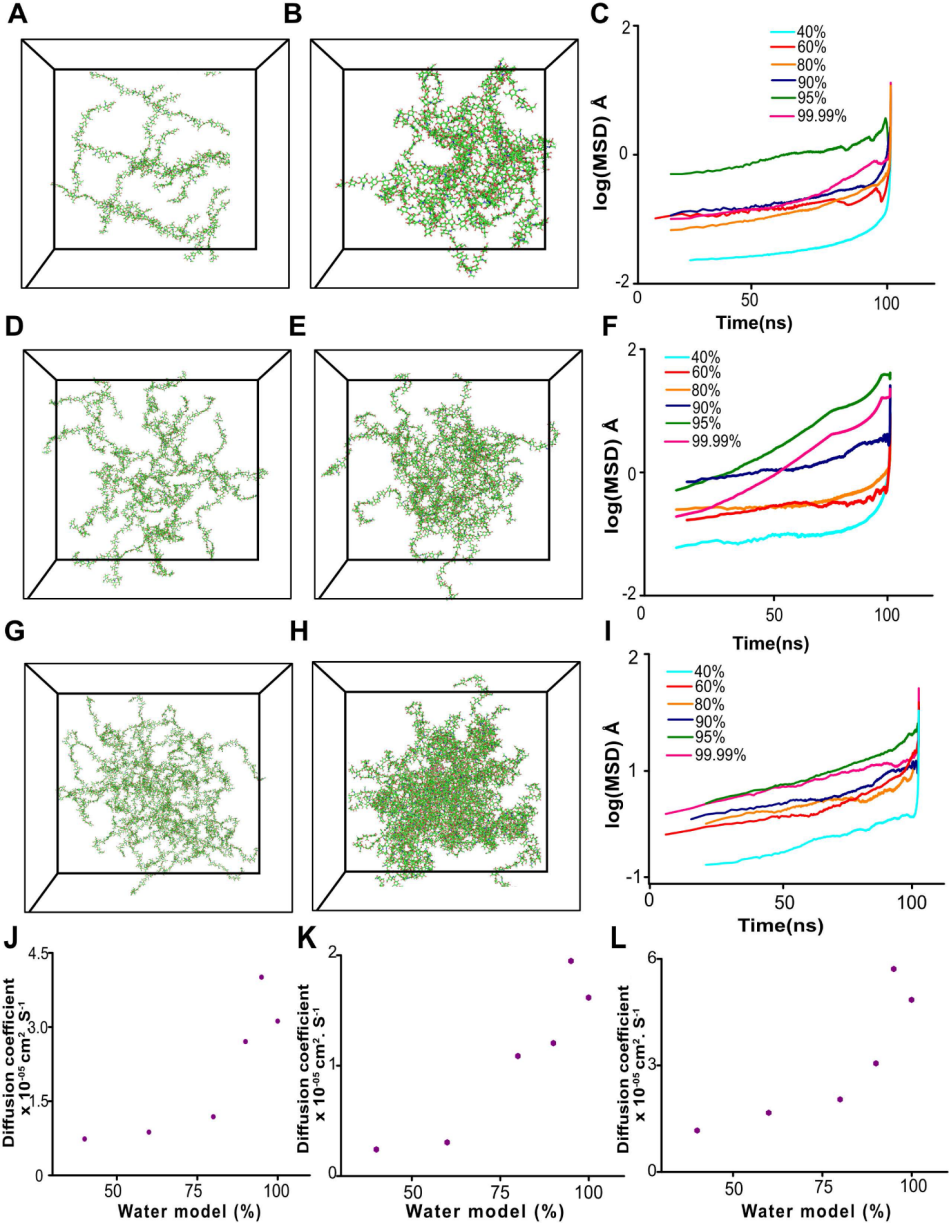
Molecular mobility analysis in HA hydrogels (mean square displacement [MSD]). (A, D, G) The initial (0 ns) and (B, E, H) final stable (100 ns) structures of the 16, 32, and 64-chain configurations, respectively. (C, F, I) log (MSD) curves illustrating water molecule diffusion in hydrogels with various water content systems for the 16, 32, and 64-chain configurations, respectively. (J–L). Diffusion coefficients of water molecules in HA hydrogels were obtained with various water content systems for 16, 32, and 64 chains, respectively. The percentage of water models is calculated as defined in the Molecular Dynamics Simulation Setup, and the exact atom count is listed in Table S1.

The MSD for the HA hydrogel with different numbers of HA chains (16, 32, and 64) and water contents (40–99.99%) is plotted against time in Figure 4. For 16 chains, the HA hydrogel system exhibits the highest MSD values, indicating greater molecular mobility. This suggests that with fewer chains, there is an increased amount of free volume inside the hydrogel, allowing water molecules and chains to move more freely. In contrast, the 64-chain system has the lowest MSD values, suggesting limited molecular movement due to its higher chain density, where the interactions and entanglements of the polymer chain severely constrain the mobility. Meanwhile, the 32-chain system exhibits intermediate behavior, striking a balance between confinement and mobility. These results align with previous studies where higher polymer density in hydrogels results in reduced water diffusion and chain mobility due to enhanced entanglements. This finding aligns well with prior findings indicating that polymer chain mobility may influence the diffusion of water molecules inside the gel network.^55−57^

To understand the hydrodynamics of the HA hydrogel and study the diffusion of water molecules inside the hydrogel network, the MSD of water molecules is calculated. The gradual increase in diffusion coefficients with increasing water concentration is consistent with expectations, as higher water content generally reduces the network density and enhances molecular mobility.^28^ All of the values are smaller than the bulk water self-diffusion value, which is about 5.05 × 10^–5^ cm^2^·s^–1^. It is observed that HA hydrogels, after self-assembly, could reduce the water molecules’ diffusion rate and restrict the movement of water molecules. Similarly, the ability to constrain water molecules is also weakened when the water content rises because the cross-linking between hydrogels becomes weaker and the distance between the molecular chains increases. In the case of higher water model systems (95 and 99.99%), the self-diffusion coefficient values decrease; it could be attributed to additional factors inherent to the hydrogel system. At 95% water model systems, the HA hydrogel structure may undergo modest reconfiguration, resulting in localized regions of enhanced density or entanglement. Despite the overall higher water concentration, these structural alterations may restrict the movement of diffusing molecules, lowering the diffusion coefficient. This is clear from the H-bond analysis, which demonstrates that the 95% system has a higher number of intermolecular H-bond formations than the 99.99% system. These results align with earlier findings, which suggest that hydrogen bond formation or dipolar molecular interactions between the water molecules and polymer chains create a high degree of hydration, which might restrict the water molecule movement in hydrogels by creating a cross-linked polymer chain network resulting in a swollen hydrogel structure with a low diffusion coefficient.^47,58,59^ Nevertheless, the interactions inside the hydrogel matrix may become more complex as a result of the increased number of chains, producing nonlinear behaviors in the MSD. In conclusion, the MSD analysis shows a relationship between the hydrogel’s structural properties and its dynamic behavior at various chain configurations and hydration levels. All chain configurations show a gradual increase in MSD with increasing water content, indicating the crucial role that hydration plays in promoting molecular mobility within the hydrogel. The results of 16, 32, and 64 chains provide insight into how structural differences affect the diffusion properties, which could help in the design and improvement of hydrogel-based systems for a range of uses.

#### Structural Organization of HA Hydrogels: Insights from Radial Distribution Function Analysis

**Figure 5:**
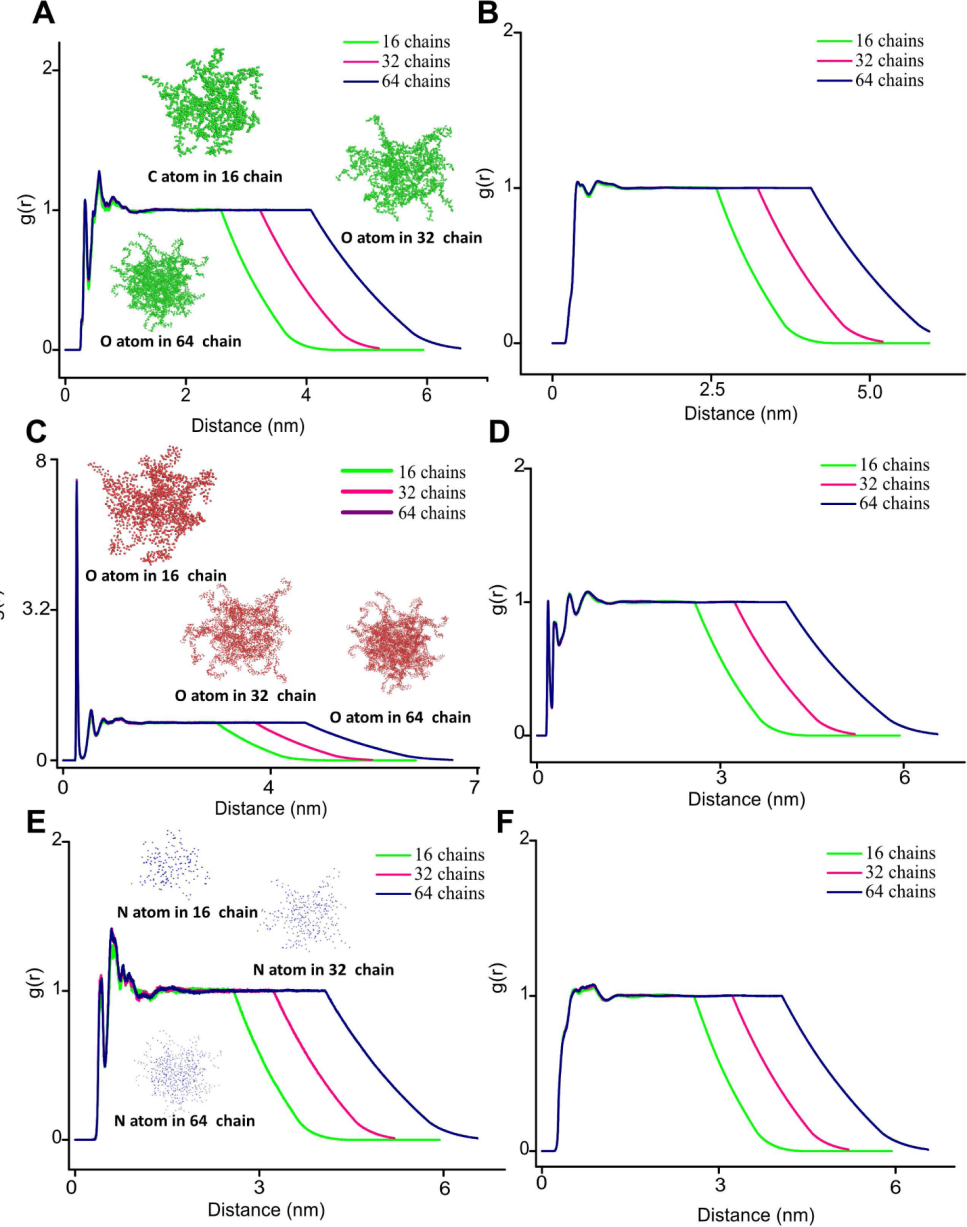
Evaluating HA hydrogel structure through radial distribution function (RDF) for the higher water model (99.99%). RDF analysis of carbon (C), oxygen (O), and nitrogen (N) atoms of hydrogels with sodium ion (Na^+^). (A, C, and E) Na^+^ ions and C, O, and N atoms of 16, 32, and 64-chain configurations, respectively. (B, D, and F) RDF analysis of water molecules and C, O, and N atoms for the 16-, 32-, and 64-chain configurations, respectively. The inside images show the distribution of C, O, and N atoms for hydrogels with different HA content.

To understand the structural organization and spatial arrangement of HA atoms in the hydrogel configuration, we examined the radial distribution function (RDF), which shows how the density of atoms or molecules changes with distance from a reference atom or molecule.^28^Figure 5 shows the higher (99.99%) water model plot for the carbon (C), oxygen (O), and nitrogen (N) atoms of HA hydrogel with sodium ions (Na^+^) and C, O, and N atoms of 16, 32, and 64-chain HA hydrogel with water molecules, and Figure S10 shows the RDF plots for remaining water content models (40–99%). There are noticeable peaks in the RDF plots (Figure 5A,C,D) for the C, O, and N atoms in the HA hydrogel system, suggesting the existence of distinct local structures. The presence of nearest neighbors at close distances is indicated by the RDF for C atoms, which displays a strong peak about 0.1–0.5 nm and a gradual reduction after that. A smaller peak emerges around 0.5 nm, while the initial peak occurs at about 0.3 nm. The RDF around C and N atoms exhibits a rapid peak followed by a decline, but it differs slightly in peak height and position. The RDF for O atoms shows a predominant peak around 0.1 nm, which suggests high local ordering in the HA hydrogel structure. This result aligns with earlier reports that carboxylate groups form a specific interaction with Na^+^, favoring compact chain conformation and localized clustering. VdW forces, with a prominent peak at 0.46 nm, are the main interaction between the O atom and Na^+^. The hydrogen bonds between these atoms are also indicated by a comparatively high peak at 0.31 nm. In general, the peaks around 0.35–0.5 nm are due to the vdW interactions, while the peaks within 0.35 nm are generally formed by hydrogen bonds and chemical bonds.^28^ It indicates that hydrogen bonding and vdW interactions are the most important interactions between the two types of atoms. Despite the presence of a weaker vdW force,^28^ these results align well with our QM results.

The behavior of the RDF remains constant at varying concentrations. A peak at 0.19 nm and two smaller peaks at 0.23 and 0.37 nm are shown in Figure 5E. The sharp peak observed in the RDF for the O atoms in the HA hydrogel, calculated against Na^+^ ions, indicates strong and well-defined interactions between the Na^+^ ions and the O atoms, likely due to ion–dipole interactions. This sharp peak suggests a high local density of Na^+^ ions at a specific distance from the O atoms, which are typically part of the carboxyl or hydroxyl groups in the HA structure. The Na^+^ ions are attracted to the negatively charged O atoms, forming ion pairs or structured hydration shells. In contrast, the RDF for C and N atoms shows weaker or broader peaks, as the interactions between Na^+^ ions, and these atoms are generally less pronounced due to the lower electrostatic attraction compared to that of O. This observation reflects the highly structured and localized nature of the Na^+^–O interactions in the HA hydrogel system. On the other hand, as the water content increases, the total height of the curve reduces. With an increasing chain count, the C system exhibits a more structured arrangement, as seen by a peak that stabilizes following the initial rise.

Relative to the 16-chain system, the RDF for the N system has the same characteristics, but its peaks are less prominent, indicating a more distributed local environment. Furthermore, even at larger chain counts, the O system retains its high initial peak and exhibits a significant propensity to develop local structures, which aligns with our QM results. The RDF patterns become smoother over the 64 chains, indicating a more uniform atom distribution. A steady peak that gradually broadens in the RDF for C atoms indicates a wider variety of atom-to-atom distances. On the other hand, the peaks for N atoms are not sharp, suggesting that local ordering decreases as chain count increases. When considering the RDF in the presence of water molecules, a notable peak in the 0.2–0.3 nm range indicates that nonbonding interactions among O and water molecules are dominated by hydrogen bonding (Figure 5B,D,F). In all the figures, we observed high peaks of about 0.2 nm, suggesting that the hydroxyl groups in HA interacted with water molecules through hydrogen bonds. Overall, the interactions between N atoms and water molecules are rather weak, but the interactions between C and O atoms are quite strong, significantly contributing to the stability of the gel network structure.^60^ The peaks in the RDFs tend to get broader and less pointed as the HA chains get longer, suggesting that the local structure gets less defined and more scattered. Based on these data, it seems that the HA hydrogel preserves some degree of local ordering, particularly for the O atoms, even at various concentrations and chains of HA. At lower chain counts and greater water concentrations, the whole structural integrity and local interactions become more noticeable.

#### Short-Range Interactions in Hyaluronic Acid Hydrogels: Analysis of Lennard–Jones and Coulomb Potential Energies

Hydrogel formation is significantly influenced by the electrostatic effect. To better understand the significance of positively charged ions in hydrogel formation, we calculated the electrostatic interaction energy (Coulomb potential) and van der Waals interactions (Lennard–Jones potential).^3,54,61^ A branching network structure forms in the HA molecules as a result of Na^+^ ions forming weak coordination with two O atoms of carboxyl groups due to electrostatic attraction. The analysis of Coulomb short-range (SR) and Lennard–Jones (LJ) potential energy calculations for HA hydrogels with 16, 32, and 64 chains (Figures S11 and S12) gives insight into the system behavior for salt concentrations and varied hydration levels. Figure S11 A,C,D shows the LJ potential energy calculation for Na^+^ ions and C, N, and O atoms in 16, 32, and 64 chains systems. Figure S12C,D shows the Coulomb SR potential energy calculation for Na^+^ ions and C, N, and O atoms in 16, 32, and 64-chain systems. The electrostatic interactions within HA hydrogel structures are identified through the Coulomb SR potential energy graphs. While the total number of chains increases from 16 to 64, we notice stronger overall interactions, as evidenced by an increase in potential energies for all atom types. The LJ potential energy data, which also demonstrates more prominent impacts in the 64-chain system, corresponds with this trend. Both the Coulomb SR and LJ potentials for C atoms show notable variations, particularly at lower hydration levels.

At various hydration levels, the behavior of N atoms is comparatively steady in both the LJ and Coulomb SR potentials. This suggests that N atoms maintain stable interactions inside the hydrogel matrix, presumably due to their participation in hydrogen bonds or other stabilizing interactions. When hydration levels increase, the O atoms exhibit the most notable alterations in both potential energy computations. This behavior indicates that O atoms are important for intermolecular interaction between the HA chains. With an increase in the number of chains from 16 to 64, both potential energy calculations show more complex and tangled connections. The 32 and 64-chain systems exhibit more overlapping and condensed energy distributions, whereas the 16-chain system exhibits a more prominent division between the various hydration levels. This implies that more complex interaction networks exist in bigger systems, maybe as a result of water-mediated effects and interchain interactions. All systems show that potential energies stabilize at higher hydration levels, indicating that the hydrogel becomes more equilibrated with an increasing water content.

## Discussion

To investigate the structural stability and integrity of HA molecules and the dynamic nature of HA chains and HA hydrogels, we used a comprehensive computational approach that combined QM and MD simulations. To achieve a balance between computing efficiency and accuracy, our multiscale approach strategically employs various computational techniques. QM studies for disaccharide units observe the stable geometry and molecular interactions surrounding the HA molecules, providing an overview of the fundamentals of the HA polymer’s building blocks (Figure 1). To prepare the computational model of the short- and long-chain HA and HA hydrogel preparation, we switch to MD simulations. The use of MD in modeling larger systems for longer time scales is crucial to accurately represent the collective behavior of polymer HA chains and their interactions with water molecules during hydrogel preparation. By combining QM and MD, we can establish a connection between atomic-level interactions and macroscopic features, offering a more comprehensive understanding of the behavior of HA, ranging from individual molecules to intricate hydrogel networks.

ESP analysis provides information about the charge distribution on molecular surfaces by highlighting the negative and positive potential regions around the molecules. These highlighted regions correspond to molecular interaction sites that govern how disaccharides interact with other polymer chains or other molecules and influence the structural integrity and dynamics of the HA hydrogel. The RDG analysis clearly explains the types of interactions between HA disaccharides, such as van der Waals interactions and hydrogen bonding. These interactions are crucial for understanding the strength and nature of the forces holding the disaccharides together. Our results agree with previous studies and suggest that the hydrogen bonds and dipolar molecular interactions between the water molecules and polymer chains can affect the diffusion of solutes in hydrogels by creating a network of cross-linked polymer chains that restrict the movement of molecules.^46,59^ The observed electrostatic potential energy surface map elucidates the charge distribution, molecular diffusion, and molecular interaction sites that govern how disaccharides provide insights into molecular diffusion and rearrangement within the hydrogel, with implications for drug delivery applications.^6,46,47^

The RDF analysis for all HA hydrogel systems reveals a sharp peak for the O atoms, which exhibits a constant and frequent involvement in hydrogen bonding. This result is further supported by ESP analysis in QM, which states that negative charges are enveloped on the O atoms of HA due to their higher ED, making them key sites for hydrogen bond acceptance. For all the systems, we notice a general pattern of larger RMSF values at the terminal residues, indicating more flexibility at the end terminal regions of the HA polymer.^13^ The Rg results highlight the crucial role of weak noncovalent interactions in maintaining hydrogel stability and reveal the significant contribution of O atoms in forming hydrogen bonds and maintaining local order (Figure 3). According to Coulomb and LJ potential energy studies, the 32- and 64-chain systems exhibit more overlapping and condensed energy distributions, whereas the 16-chain system exhibits a clearer demarcation between the various hydration levels (Figures S11 and S12). It suggests that systems with higher HA concentration have more complex networks due to interchain interactions and water-mediated effects. Increasing the hydration levels leads to a stabilization of potential energies in all systems, suggesting that the hydrogels reach a more balanced state as the water content increases. This is important for comprehending macroscale behavior and properties of HA hydrogels in a range of applications. Our systematic study of hydration levels and scaling effects offers valuable guidance for controlling hydrogel properties during fabrication processes. These findings reveal the complex interplay of interactions in HA hydrogels and can be used to generate testable predictions, hopefully contributing to rational hydrogel development. Our study focuses on an explicit water model to effectively capture water motility within the HA polymer chains, which is crucial for understanding the dynamic behavior of the hydrogels. In future studies, we plan to extend the QM analysis for longer HA disaccharides in different phases and MD simulation for a higher number of HA disaccharides or HA polymer chains and extend the simulation box size, enabling more realistic HA modeling.

## Conclusions

To better comprehend the molecular basis of the structural and functional properties of the HA chain and hydrogels, we used QM and MD simulations. Our QM findings shed more light on the chemical relationships and energy profiles of the HA chain. Strong and weak interaction regions are highlighted by analyzing the RDG distribution, which shows noncovalent interaction regions of the HA disaccharides. These QM findings elucidate the charge distribution, molecular diffusion, and molecular interaction sites that govern how disaccharides provide insights into molecular diffusion and rearrangement within the hydrogel, with implications for drug delivery applications. This method can shed light on the stability and reactivity of HA disaccharides under diverse circumstances, clarifying their dynamic behavior and performance in various applications. MD results reveal that the presence and concentration of salts substantially affect the stability of the HA hydrogels. According to our findings, there is a clear and consistent pattern whereby increasing salt concentrations causes the Rg to fluctuate more, which indicates an alteration of the hydrogel stability. These changes indicate that higher salt concentrations may compromise hydrogel stability, resulting in a more dynamic and less rigid network topology. To verify the accuracy of our findings, we ran many simulations under various initial circumstances and confirmed that the observed variations remained robust throughout these runs, ruling out potential modeling or computational instabilities that impact the results. Our simulation results and existing research both support the pattern, thus confirming the accuracy of our conclusions. Coulomb and LJ potential energy analyses provide additional insights into HA hydrogel behavior, highlighting the complex correlations between chain counts and hydration. In summary, these outcomes provide valuable insights into the HA hydrogel formation mechanisms and guidelines for hydrogel design and development. To that extent, our code is available open source on GitHub at https://github.com/Synthetic-Physiology-Lab/Gromacs_setup_and_polymer_analysis.

## Supporting Information Available

DFT optimized structures of HA_1–3_ and HA_1–4_ disaccharides with β_1–3_ linkage β_1–4_ linkage, 500 ns conformer structure of HA_4_, HA_6_, and HA_10_ repeated disaccharides of HA polymer at 0.1 M concentration and its RMSD, Rg, and RMSF analysis. Schematic representation of hydrogel formation, RDF analysis of sodium ions and water molecules around HA hydrogel, the short-range LJ and Coulomb potential energy graph, inter and intramolecular hydrogen bond formation. Table of water molecules added to the different numbers of HA hydrogel systems (PDF)
