# Supplementary material for: Molecular Dynamics Simulation of Structural Assembly and Hydration of Hyaluronic Acid in Salt Aqueous Buffer
Source: Langmuir. 2025 Feb 6;41(6):3852–64. doi: 10.1021/acs.langmuir.4c03966 (PMC11841034; doi:10.1021/acs.langmuir.4c03966)
Supplement: Supplementary file 1 — la4c03966_si_001.pdf [file la4c03966_si_001.pdf]

Supporting Information

Molecular Dynamics Simulation of Structural Assembly and Hydration of Hyaluronic Acid in Salt Aqueous Buffer

Saranya Vasudevan[a,*], Sandipan Chattaraj[a], Alessandro Enrico[a], Francesco Silvio Pasqualini[a,*]

[a] Synthetic Physiology Lab, Department of Civil Engineering and Architecture, University of Pavia, Pavia, 27100, Italy

* co-corresponding authors: francesco.pasqualini@unipv.it, drsaranyavasudevan.phy@gmail.com

List
Number of pages: 14
Number of figures: 12
Number of tables: 1

Table of Contents

Figure S1. Chemical structure of (A) HA_{1-3} and (B) HA_{1-4} disaccharides with β_{1-3} linkage $_{\beta 1-4}$ linkage.
Figure S2. (A) RMSD, (B) 500 ns conformer structure, (C) Rg, and (D) RMSF for HA_4, HA_6, and HA_{12} repeated disaccharides of HA polymer at 0.1 M concentration.
Figure S3. The radius of gyration (Rg) for 20 repeated disaccharides of HA (HA_{10}) at (A) charge neutralised, (B) 0.1 M, (C) 0.5 M and (D) 1 M concentrations.
Figure S4. Mean Rg along with standard deviation of HA_6 at (A) $CaCl_2$, (B) NaCl, and (C) KCL system for different concentrations.
Figure S5. Distribution of cations around the HA_6 at (A) charge-neutralized state, (B) 0.1 M, (C) 0.5 M, and (D) 1 M concentrations.
Figure S6. Distribution of anions around HA_6 at (A) 0.1 M, (B) 0.5 M and (C) 1 M concentrations.
Figure S7. RMSF for six repeated HA disaccharide units (HA_6) at (A) charge-neutralized state, (B) 0.1 M, (C) 0.5 M, and (D) 1 M concentrations.
Figure S8. Schematic representation of HA hydrogel formation.
Figure S9. The (A) H-bond between the HA chains and (B) H-bonds between 16, 32, and 64 chains of HA and water molecules in various water content models (See also Table S1).
Figure S10. Evaluating HA hydrogel structure through Radial distribution function (RDF) with varying water models.
Figure S11. The Lennard Jones short-range potential (LJ-SR) energy of (A, B, and C) Na^+ ion and C atoms, (D, E, and F) Na^+ ions and N atoms, and (G, H, and I) Na^+ ion and O atoms of hydrogels composed of 16, 32 and 64 chains of HA, respectively.
Figure S12. The short-range Coulomb short-range potential energy of (A, B, and C) Na^+ ion and C atoms, (D, E, and F) Na^+ ions and N atoms, and (G, H, and I) Na^+ ion and O atoms of hydrogels composed of 16, 32 and 64 chains of HA, respectively.
Table S1 - Number of water molecules added to the different numbers of HA hydrogel systems.

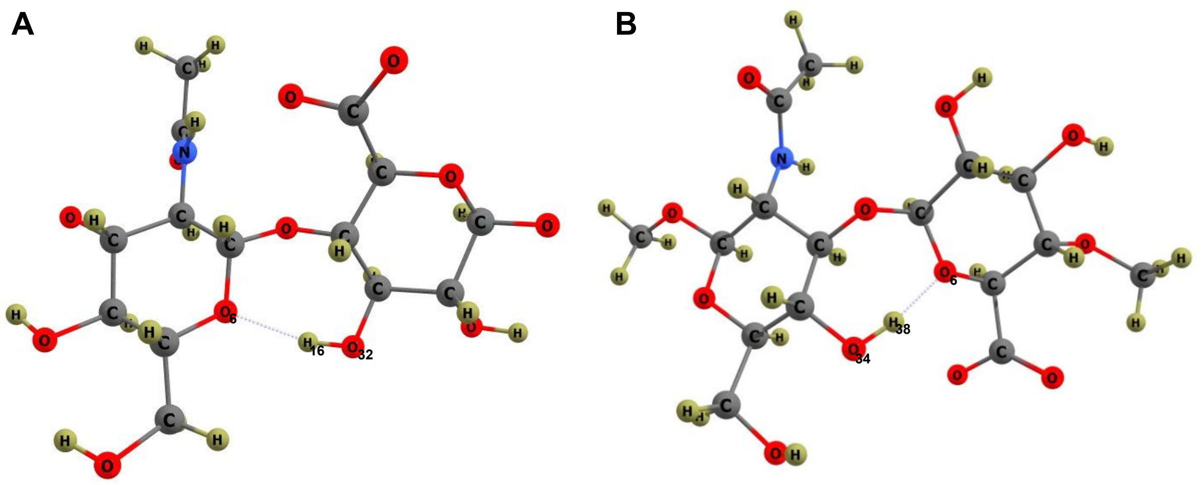

Figure S1. Chemical structure of (A) HA_{1-3} and (B) HA_{1-4} disaccharides with β_{1-3} linkage and β_{1-4} linkage, respectively.

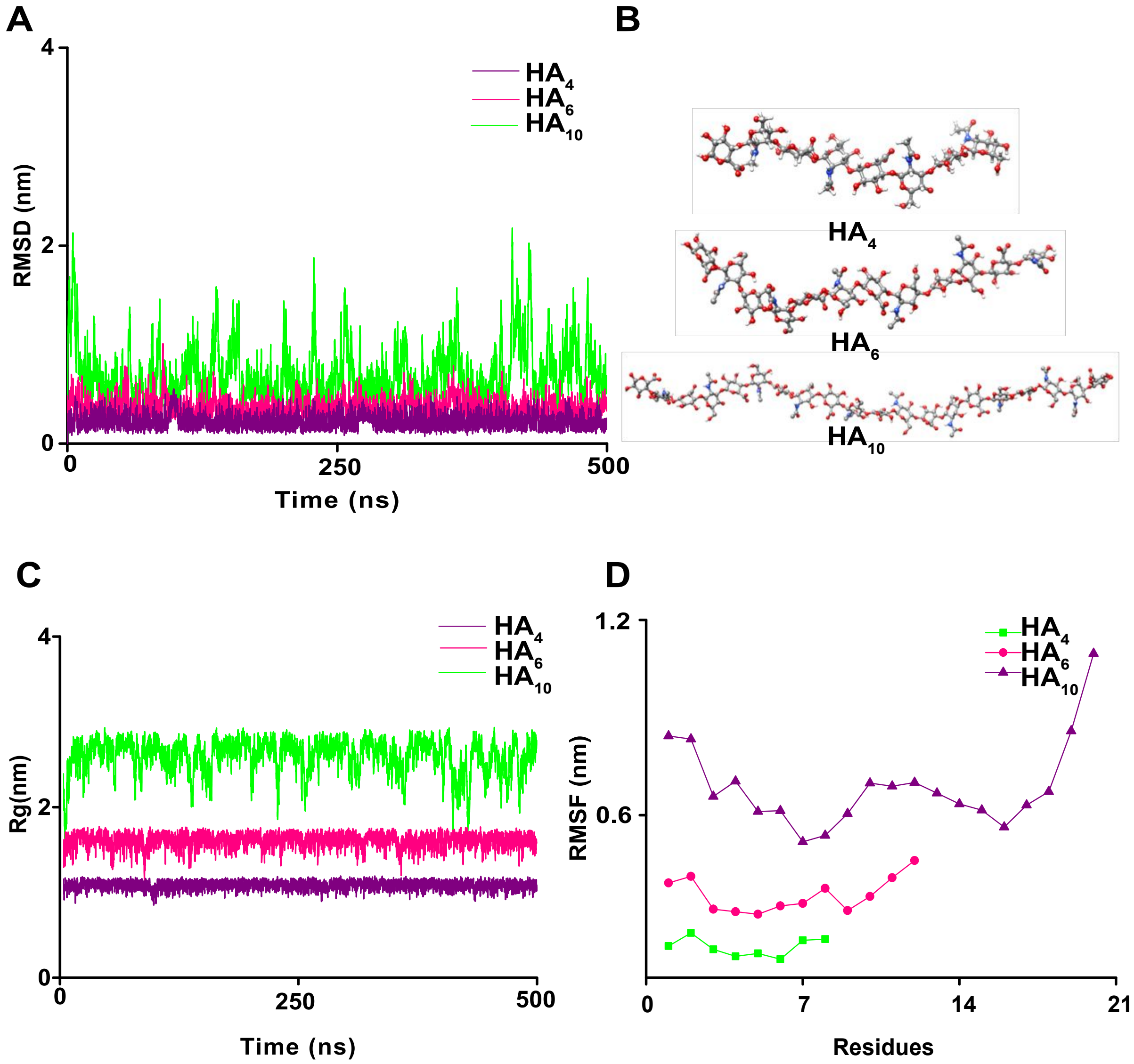

Figure S2. (A) RMSD, (B) 500 ns conformer structure, (C) Rg, and (D) RMSF for HA_4, HA_6, and HA_{12} repeated disaccharides of HA polymer at 0.1 M concentration.

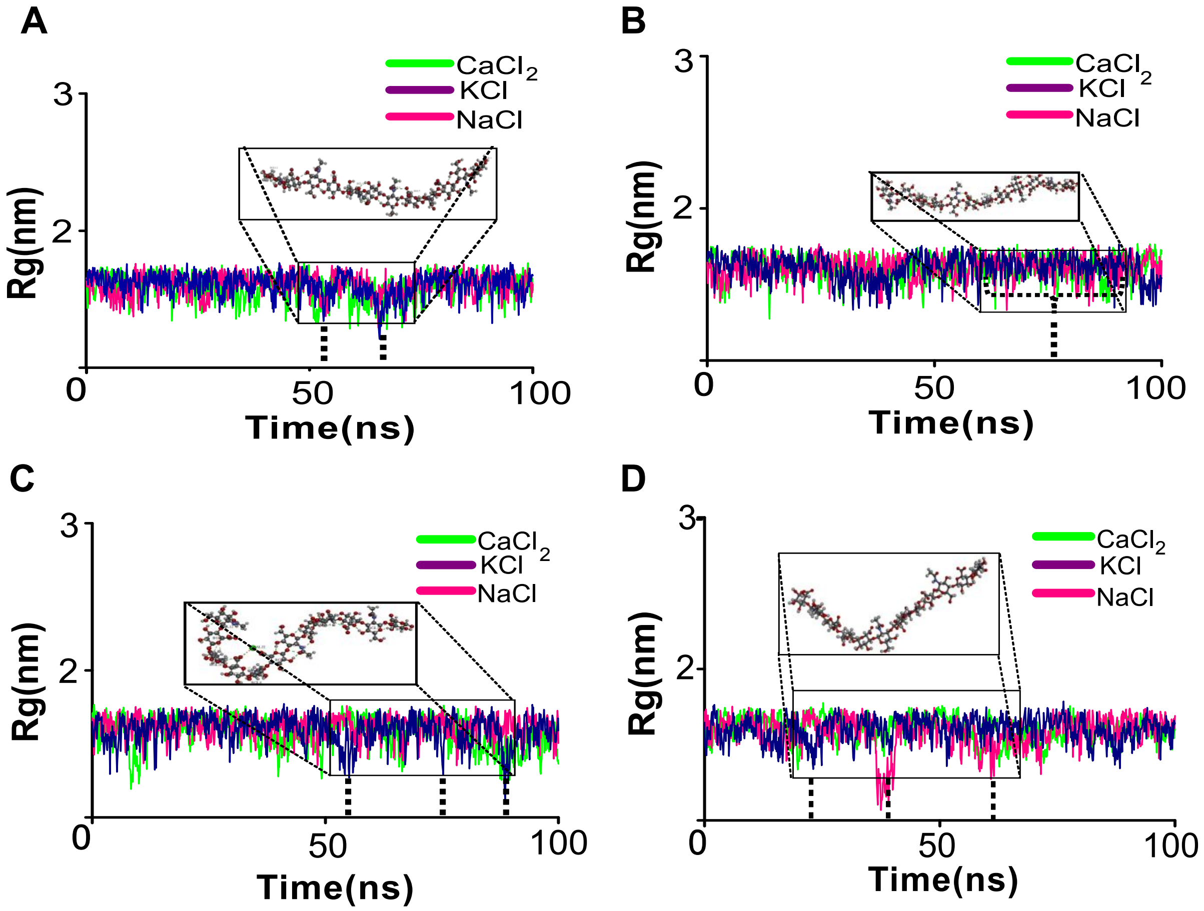

Figure S3. The radius of gyration (Rg) for 20 repeated disaccharides of HA (HA_{10}) at (A) charge neutralised, (B) 0.1 M, (C) 0.5 M and (D) 1 M concentrations. The highlighted images depict the most stable (B and D) and most unstable (A and C) structures, along with their respective regions.

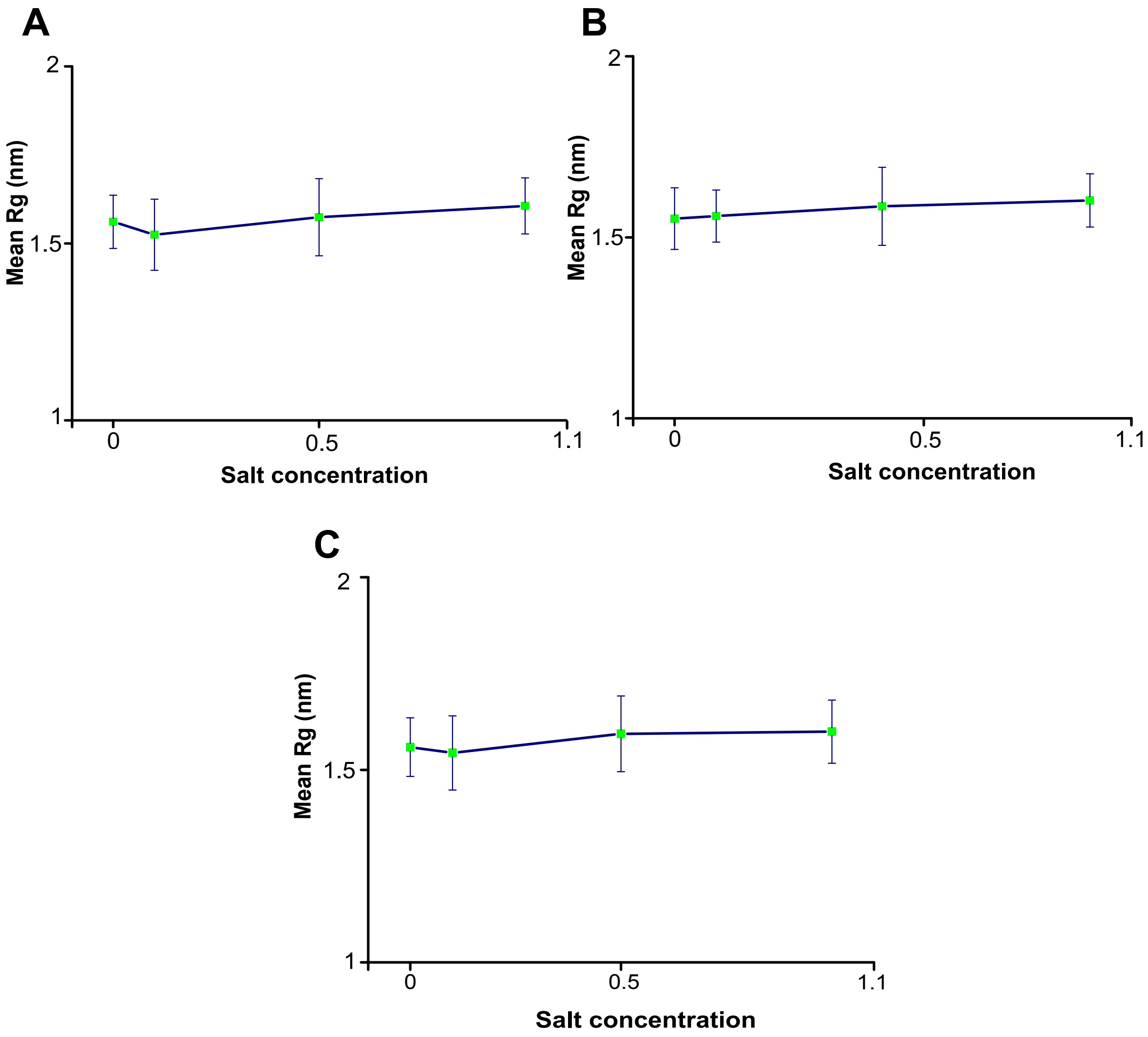

Figure S4. Mean Rg along with standard deviation of HA_6 at (A) $CaCl_2$, (B) NaCl, and (C) KCL system for different concentrations.

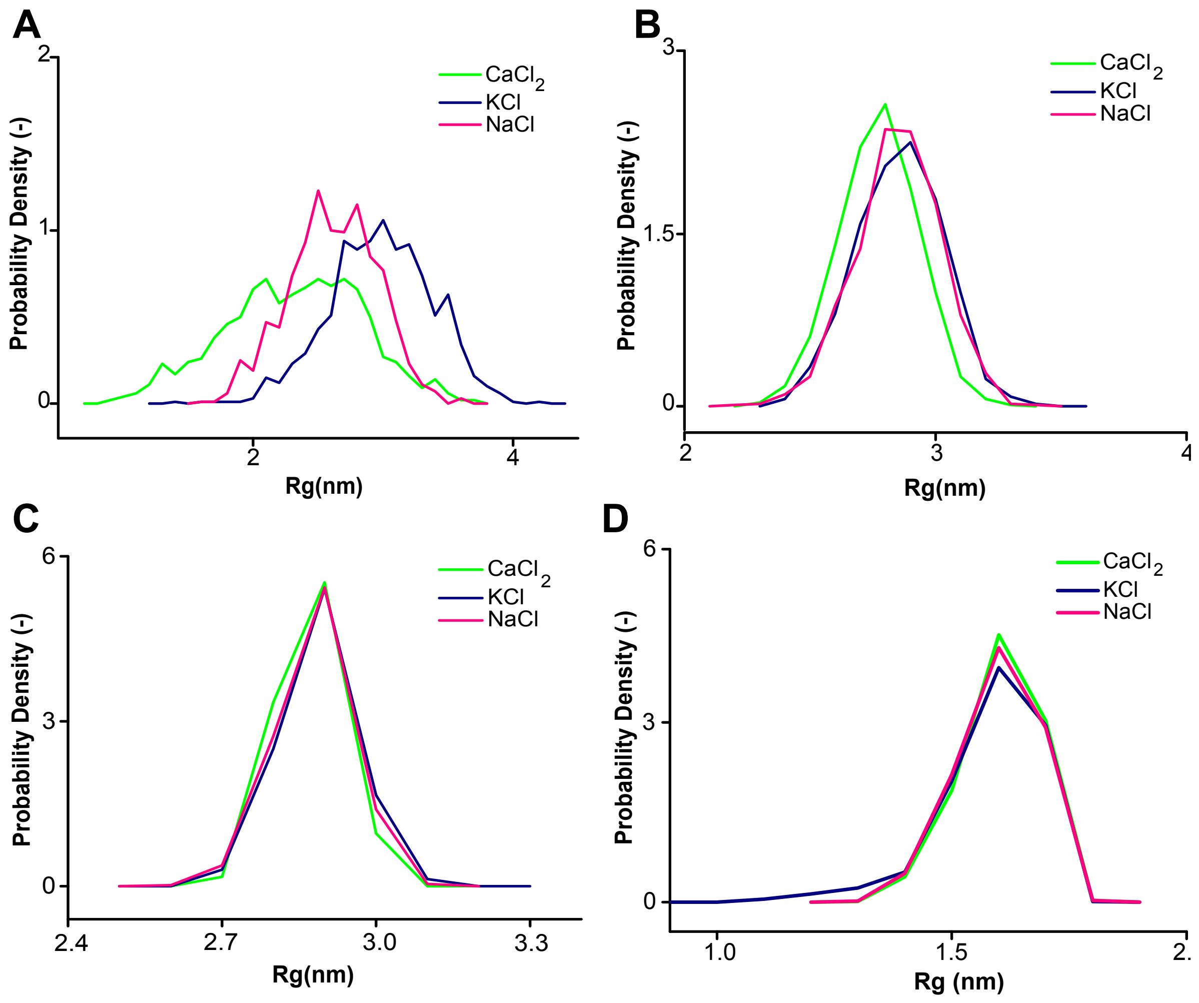

Figure S5. Distribution of cations around the HA_6 at (A) charge-neutralized state, (B) 0.1 M, (C) 0.5 M, and (D) 1 M concentrations.

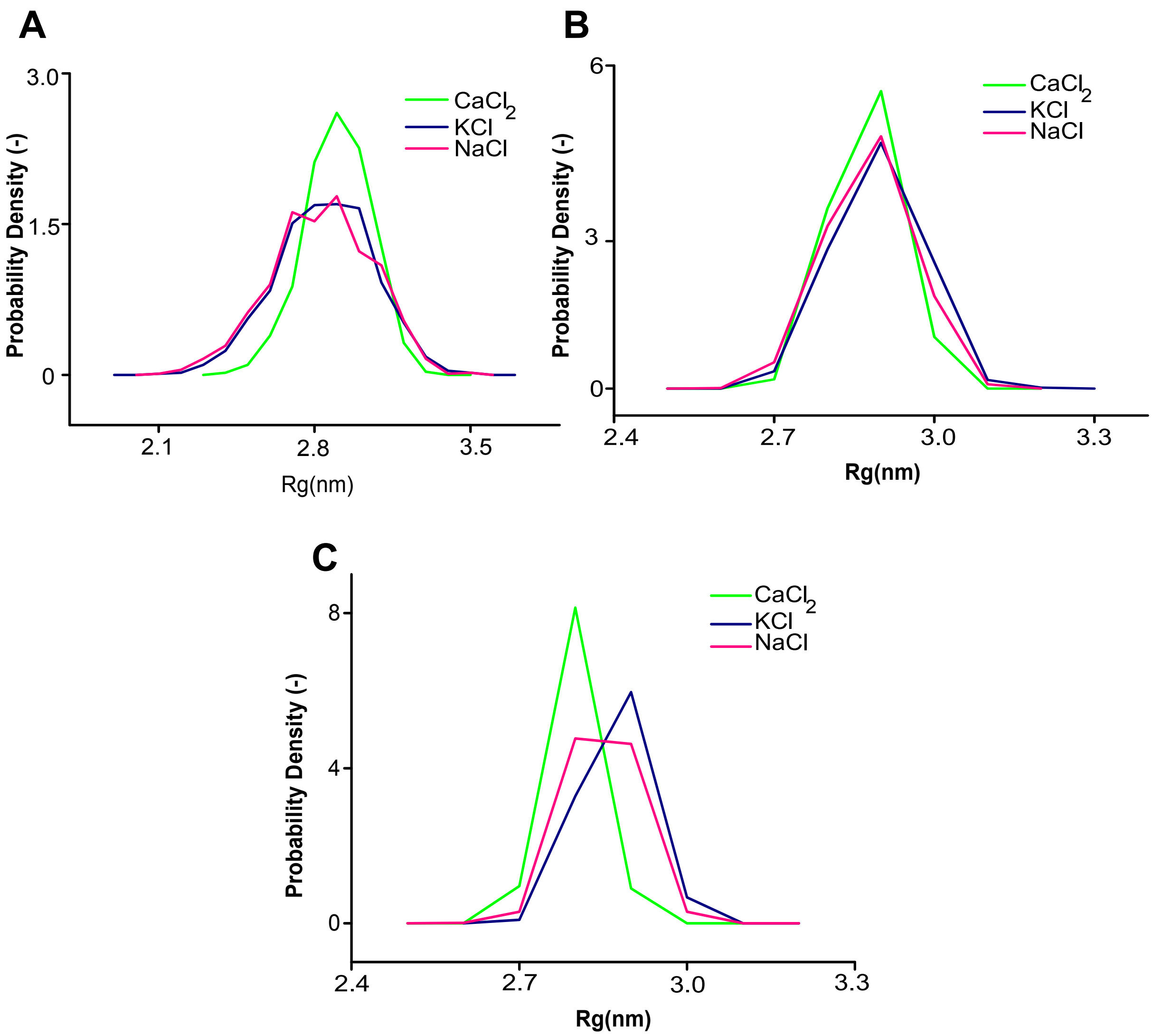

Figure S6. Distribution of anions around HA_6 at (A) 0.1 M, (B) 0.5 M and (C) 1 M concentrations.

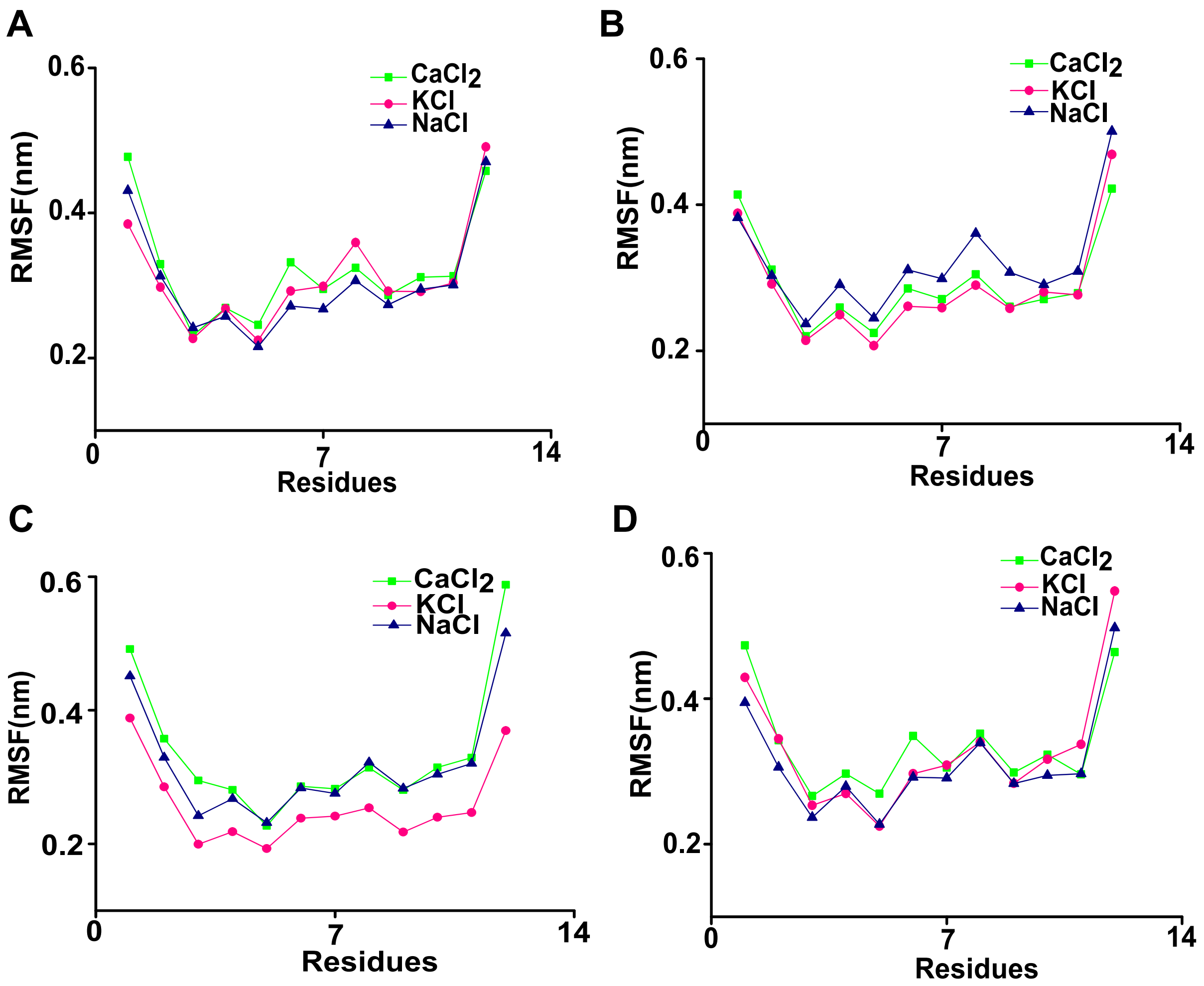

Figure S7. RMSF for six repeated HA disaccharide units (HA_6) at (A) charge-neutralized state, (B) 0.1 M, (C) 0.5 M, and (D) 1 M concentrations.

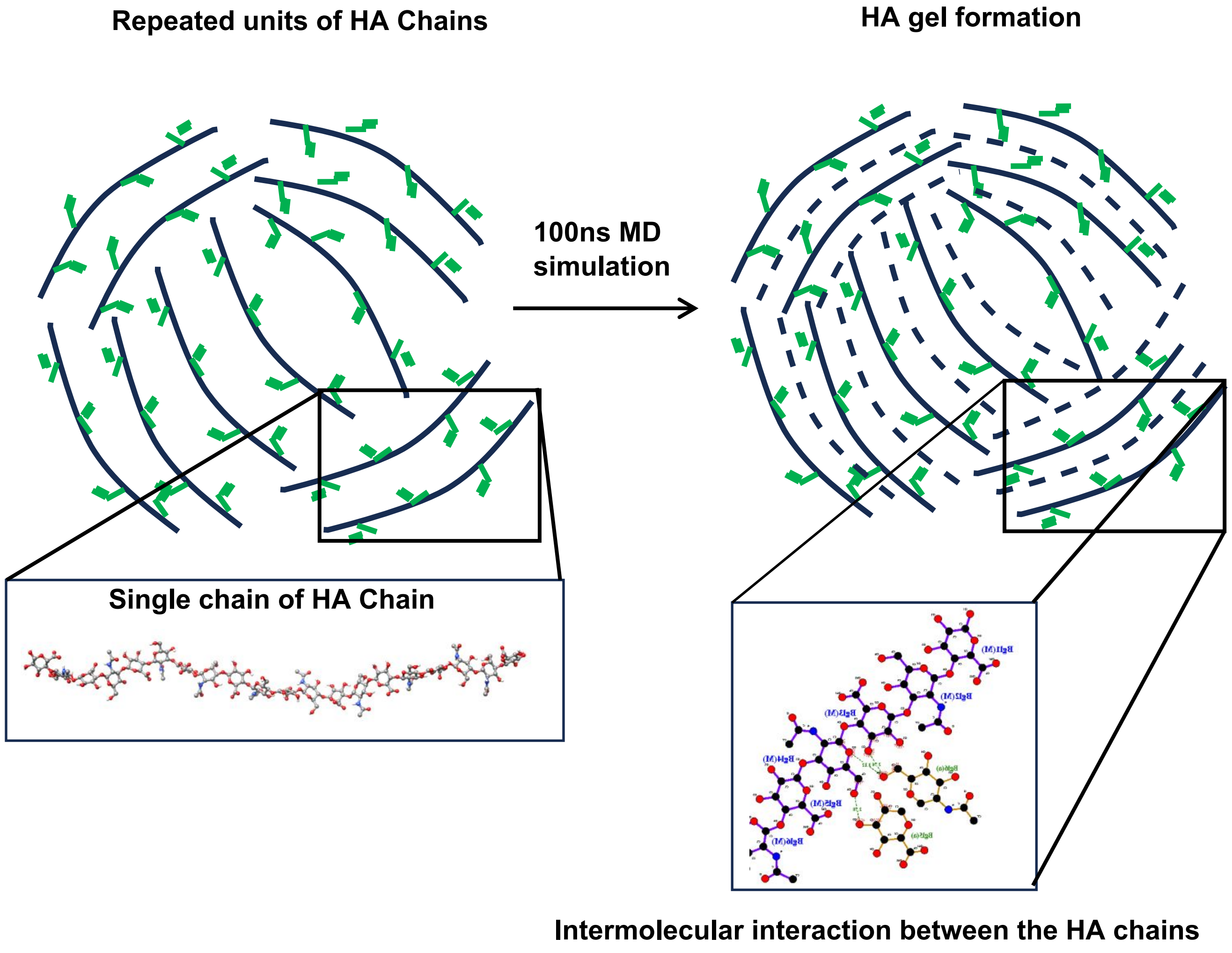

Figure S8. Schematic representation of HA hydrogel formation.

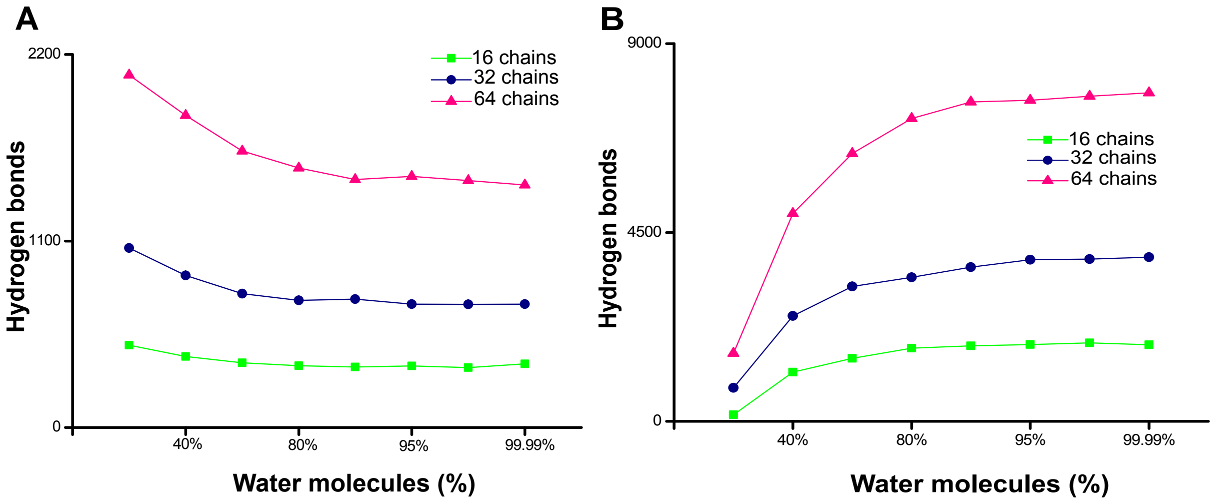

Figure S9. The (A) H-bond between the HA chains and (B) H-bonds between 16, 32, and 64 chains of HA and water molecules in various water content models (See also Table S1).

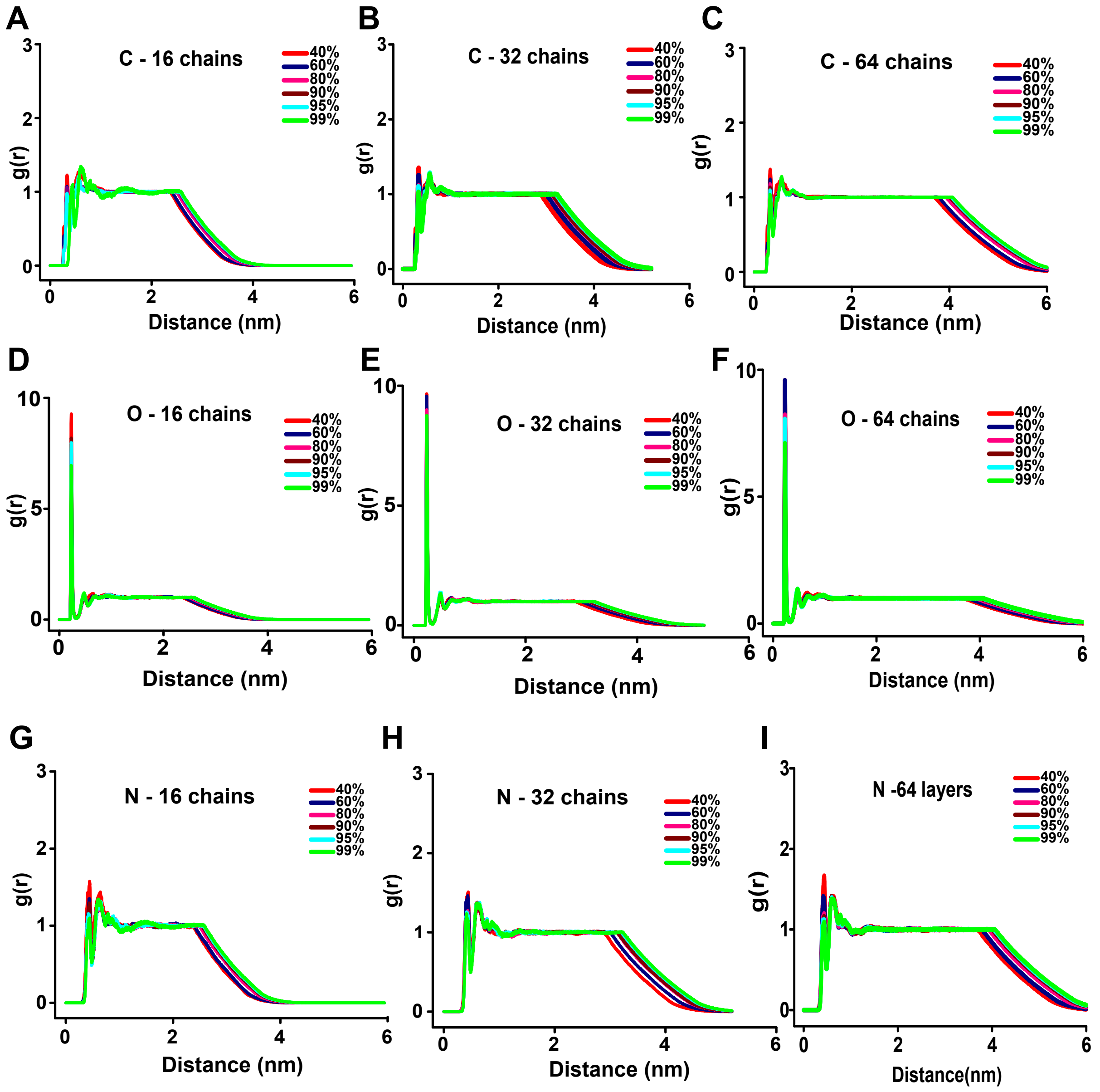

Figure S10. Evaluating HA hydrogel structure through Radial distribution function (RDF) with varying water models. RDF analysis of (A, B, and C) Na^{+} ions and C atoms of HA hydrogel in 16, 32, and 64 chains of HA hydrogel, (D, E and F) Na^{+} ions and O atoms of HA hydrogel in 16, 32, and 64 chains of HA hydrogel and (G, H and I) Na^{+} ions and N atoms in 16, 32, and 64 chains of HA hydrogel.

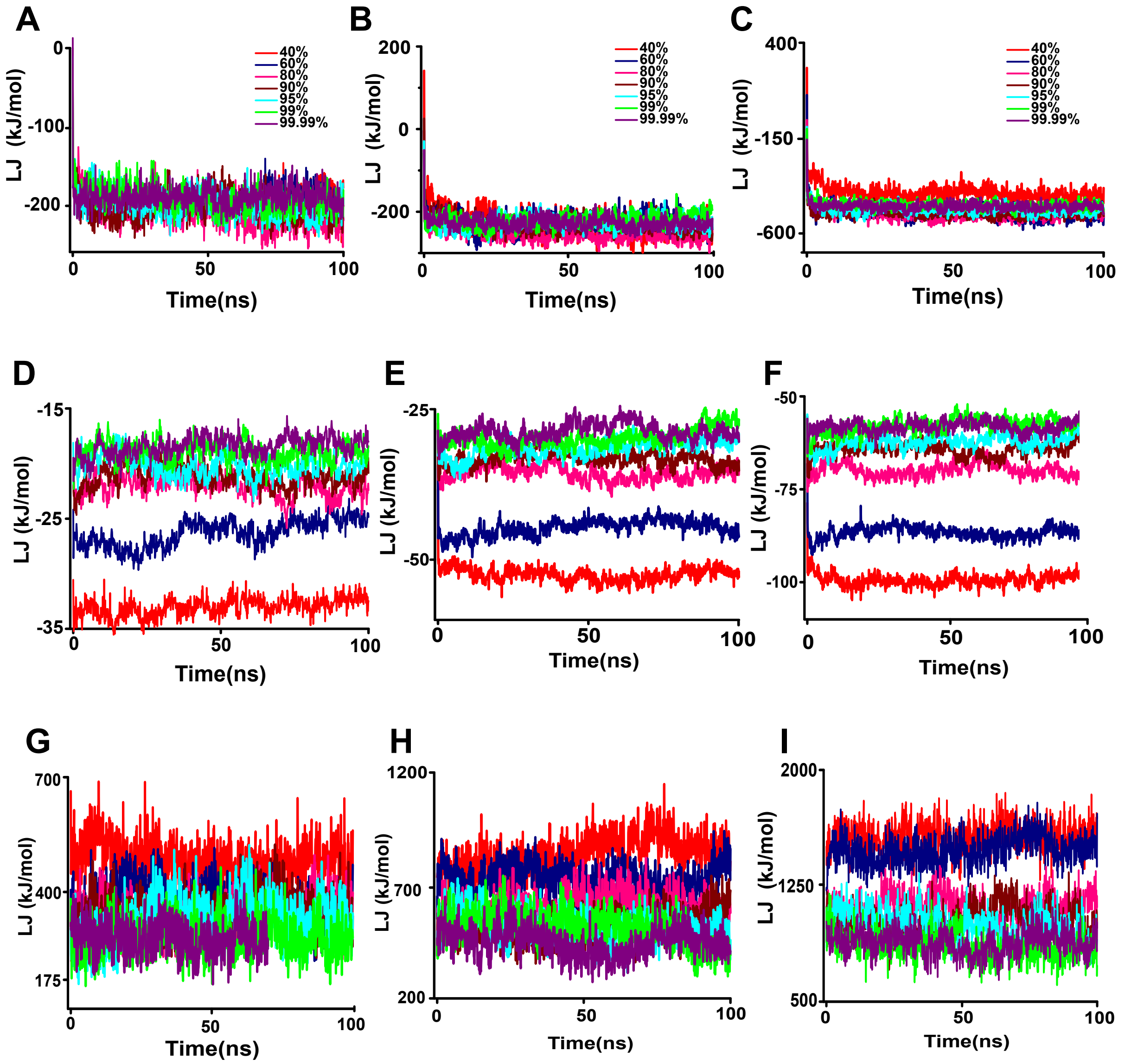

Figure S11. The Lennard Jones short-range potential (LJ-SR) energy of (A, B, and C) Na^+ ion and C atoms, (D, E, and F) Na^+ ions and N atoms, and (G, H, and I) Na^+ ion and O atoms of hydrogels composed of 16, 32 and 64 chains of HA, respectively.

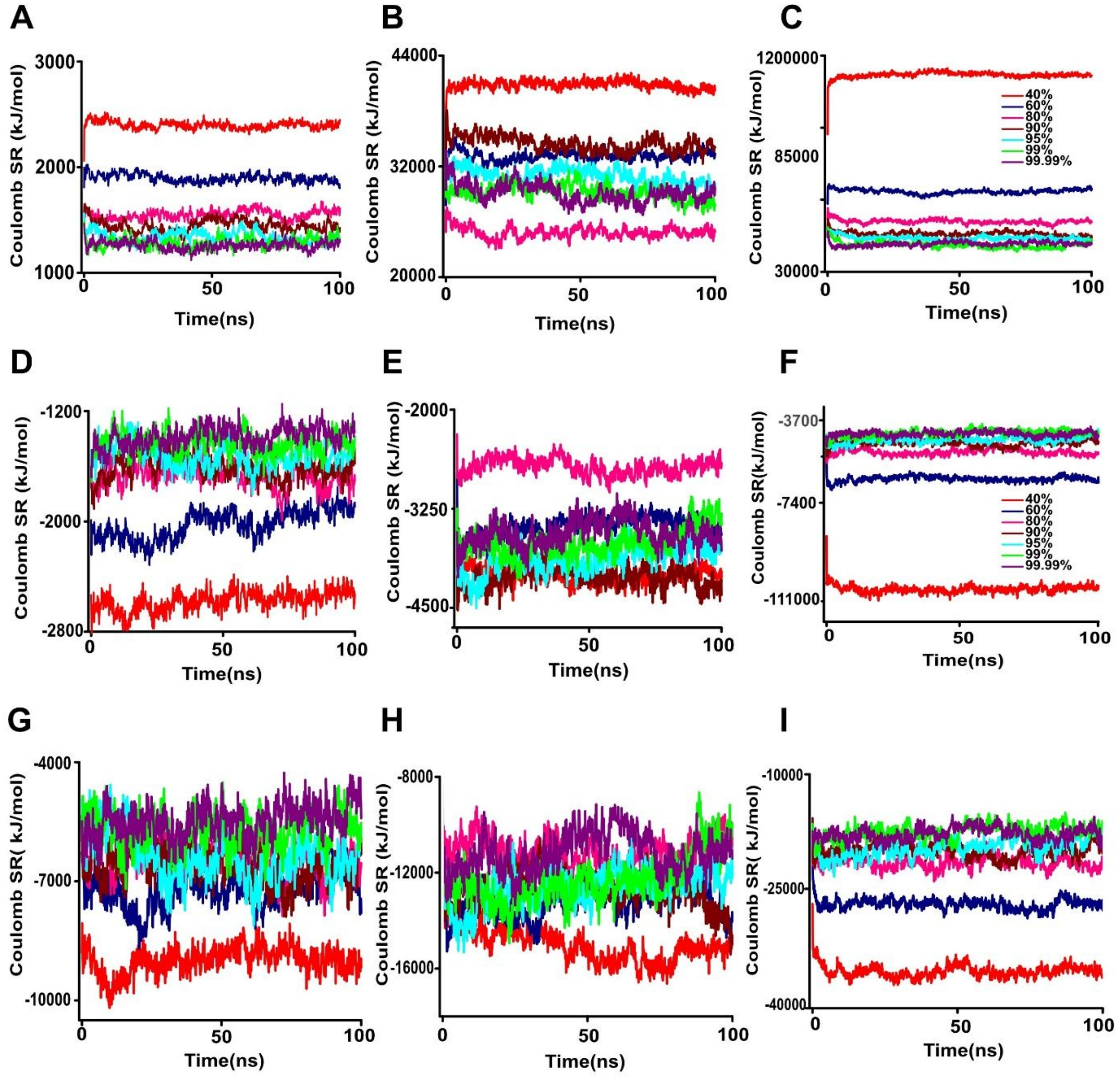

Figure S12. The short-range Coulomb short-range potential energy of (A, B, and C) Na^+ ion and C atoms, (D, E, and F) Na^+ ions and N atoms, and (G, H, and I) Na^+ ion and O atoms of hydrogels composed of 16, 32 and 64 chains of HA, respectively.

Table S1 - Number of water molecules added to the different numbers of HA hydrogel systems.

16 chains of HA				32 chains of HA				64 chains of HA			
**Number of atoms in HA chains**	**Water model (%)**	**Number of added water molecules**	**Total number of atoms**	**Number of atoms in HA chains**	**Water model (%)**	**Number of added water molecules**	**Total number of atoms**	**Number of atoms in HA chains**	**Water model (%)**	**Number of added water molecules**	**Total number of atoms**
	10	247	8149		10	494	16298		10	988	32596
	40	988	10372		40	1975	20741		40	3951	41485
	60	1482	11854		60	2963	23705		60	5926	47410
7408	80	1975	13333	14816	80	3951	26669	29632	80	7902	53338
	90	2222	14074		90	4074	27038		90	8890	56302
	95	2346	14446		95	4692	28892		95	9383	57781
	99	2445	14743		99	4890	29486		99	9779	58969
	99.99	2468	14812		99.99	4938	29630		99.99	9876	59260
